# Major depressive disorder: hypothesis, mechanism, prevention and treatment

**DOI:** 10.1038/s41392-024-01738-y

**Published:** 2024-02-09

**Authors:** Lulu Cui, Shu Li, Siman Wang, Xiafang Wu, Yingyu Liu, Weiyang Yu, Yijun Wang, Yong Tang, Maosheng Xia, Baoman Li

**Affiliations:** 1 Department of Forensic Analytical Toxicology, School of Forensic Medicine, China Medical University, Shenyang, China; 2 Liaoning Province Key Laboratory of Forensic Bio-evidence Sciences, Shenyang, China; 3 China Medical University Centre of Forensic Investigation, Shenyang, China; 4 International Joint Research Centre on Purinergic Signalling/Key Laboratory of Acupuncture for Senile Disease (Chengdu University of TCM), Ministry of Education/School of Health and Rehabilitation, Chengdu University of Traditional Chinese Medicine/Acupuncture and Chronobiology Key Laboratory of Sichuan Province, Chengdu, China; 5 Department of Orthopaedics, The First Hospital, China Medical University, Shenyang, China

**Keywords:** Diseases of the nervous system, Cellular neuroscience

## Abstract

Worldwide, the incidence of major depressive disorder (MDD) is increasing annually, resulting in greater economic and social burdens. Moreover, the pathological mechanisms of MDD and the mechanisms underlying the effects of pharmacological treatments for MDD are complex and unclear, and additional diagnostic and therapeutic strategies for MDD still are needed. The currently widely accepted theories of MDD pathogenesis include the neurotransmitter and receptor hypothesis, hypothalamic-pituitary-adrenal (HPA) axis hypothesis, cytokine hypothesis, neuroplasticity hypothesis and systemic influence hypothesis, but these hypothesis cannot completely explain the pathological mechanism of MDD. Even it is still hard to adopt only one hypothesis to completely reveal the pathogenesis of MDD, thus in recent years, great progress has been made in elucidating the roles of multiple organ interactions in the pathogenesis MDD and identifying novel therapeutic approaches and multitarget modulatory strategies, further revealing the disease features of MDD. Furthermore, some newly discovered potential pharmacological targets and newly studied antidepressants have attracted widespread attention, some reagents have even been approved for clinical treatment and some novel therapeutic methods such as phototherapy and acupuncture have been discovered to have effective improvement for the depressive symptoms. In this work, we comprehensively summarize the latest research on the pathogenesis and diagnosis of MDD, preventive approaches and therapeutic medicines, as well as the related clinical trials.

## Introduction

Major depressive disorder (MDD), a main cause of disability worldwide, is characterized by physical changes such as tiredness, weight loss, and appetite loss. Anhedonia is a classic feature of MDD, and MDD is also accompanied by a lack of drive, sleep issues, cognitive challenges, and emotional symptoms such as guilt.^1^ The prevalence of depression is increasing yearly. About 300 million people in the world are affected by MDD, which has become one of the main causes of disability.^2^ In 2018, MDD ranked third in terms of disease burden according to the WHO, and it is predicted to rank first by 2030.^3^ Pregnant women, elderly people, children, and others have a higher incidence rate of MDD, which may be related to genetic, psychological, and social factors.^4^ Depression can be accompanied by recurrent seizures, which may occur even during remission or persist for longer than the disease itself.^5^ Pharmacological therapies for MDD can effectively control symptoms; thus, patients may experience recurrence within a short time after discontinuing medication.^6^ During recurrence, the patient experiences symptoms of low mood, loss of interest in life, fatigue, delayed thinking, and repeated fluctuations in mental state.^7^

There is a certain correlation between the occurrence of MDD and social development.^8^ A survey reported that with the development of the economy and increased life pressure, MDD has begun to emerge at a younger age, and the incidence of MDD in women is approximately twice that in men.^9^ Specifically, women are more likely to develop depressive symptoms when they encounter social emergencies or are under significant stress.^8^ Additionally, autumn and winter have been reported to be associated with a high incidence of MDD, namely, seasonal depression.^10^

The clinical symptoms of MDD include a depressed mood, loss of interest, changes in weight or appetite, and increased likelihood of committing suicide.^11^ These symptoms are also listed as the criteria for MDD in the Diagnostic and Statistical Manual of Mental Disorders (DSM-5).^12^ In addition to the criteria listed in the DSM-5, the criteria reported in the International Classification of Diseases (ICD-10) are also used to guide clinical diagnosis.^13^ However, due to the lack of characteristic symptoms and objective diagnostic evidence for MDD, identification and early prevention are difficult in the clinic.^14^

**Fig. 1:**
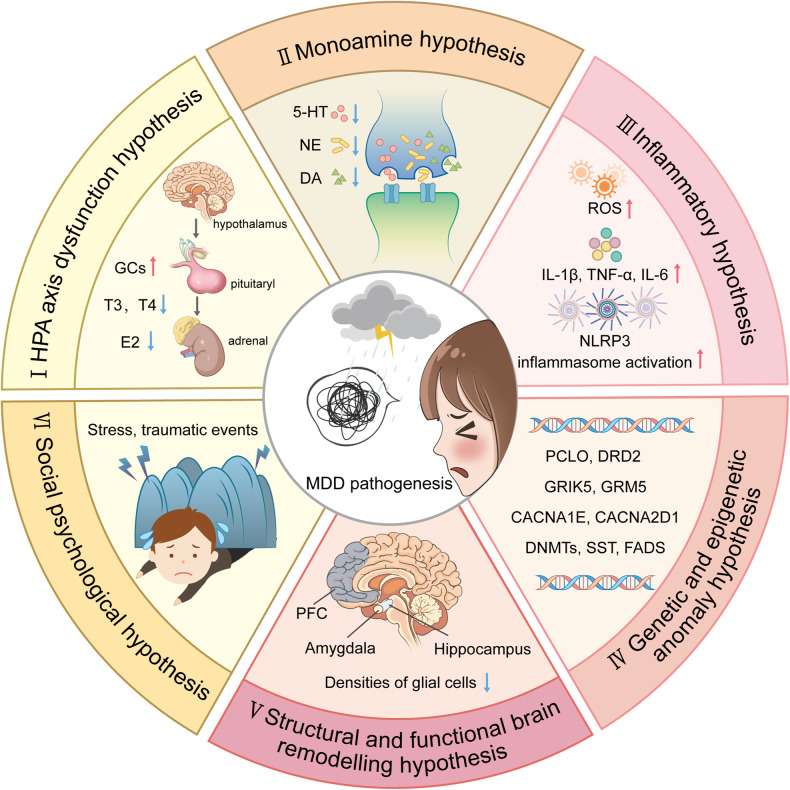
An outline map of the hypotheses to explain MDD pathogenesis. (I) HPA axis dysfunction hypothesis: high levels of glucocorticoids (GCs) play a core role in the pathogenesis of MDD, and thyroid hormone (TH) and estrogen are also involved in functions of the HPA axis; (II) the monoamine hypothesis: the functional deficiency of serotonin (5-HT), dopamine (DA) and norepinephrine (NE) are the main pathogenesis of MDD; (III) the inflammatory hypothesis: the neuro-inflammation induced by reactive oxygen species (ROS), inflammatory cytokines and inflammasomes activation is suggested to promote the occurrence of MDD; (IV) the genetic and epigenetic anomaly hypothesis: some genes are susceptible in the patients with MDD, including presynaptic vesicle trafficking (PCLO), D2 subtype of the dopamine receptor (DRD2), glutamate ionotropic receptor kainate type subunit 5 (GRIK5), metabotropic glutamate receptor 5 (GRM5), calcium voltage-gated channel subunit alpha1 E (CACNA1E), calcium voltage-gated channel auxiliary subunit alpha2 delta1(CACNA2D1), DNA methyltransferases (DNMTs), transcription levels of somatostatin (SST), fatty acid desaturase (FADS); (V) the structural and functional brain remodeling hypothesis: the postmortem results of patients with MDD are mostly associated with the reduced densities of glial cells in the prefrontal cortex (PFC), hippocampus, and amygdala; (VI) the social psychological hypothesis: the traumatic or stressful life events are the high risks of the occurrence of MDD. Adobe Illustrator was used to generate this figure

Due to the complexity of the pathological mechanism of MDD, accurate diagnostic approaches and pharmacological therapeutic strategies are relatively limited. Several hypothesis were developed to explain MDD pathogenesis pathogenic including (i) the hypothalamic‒pituitary‒adrenal (HPA) axis dysfunction hypothesis, (ii) the monoamine hypothesis, (iii) the inflammatory hypothesis, (iv) the genetic and epigenetic anomaly hypothesis, (v) the structural and functional brain remodeling hypothesis, and (vi) the social psychological hypothesis^3,15,16^ (Fig. 1). However, none of these hypotheses alone can fully explain the pathological basis of MDD, while many mechanisms proposed by these hypotheses interact with each other. In recent years, great progress has been made in identifying novel pharmacological therapies, diagnostic criteria, and nonpharmacological preventive measures for MDD, initiating related clinical trials. Specifically, increasing evidence suggests that astrocytic dysfunction plays a substantial role in MDD.^17^ Pharmacological ablation of astrocytes in the medial prefrontal cortex (mPFC) causes depressive-like symptoms in experimental animals,^18^ and postmortem studies of patients with MDD have shown reduced densities of glial cells in the prefrontal cortex (PFC), hippocampus and amygdala.^19^ In addition, glial fibrillary acidic protein (GFAP), one of the markers of astrocytes, is expressed at various levels,^20^ and the levels of connexins,^21^ glutamine synthase (GS), glutamate transporter-1 (GLT-1),^21,22^ and aquaporin-4 (AQP4)^23^ are reduced in patients with MDD.

In this review, we summarize the latest research on the etiology, pathogenesis, diagnosis, prevention, mechanism, and pharmacological and nonpharmacological treatment of MDD as well as related clinical experiments.

## Potential etiologies and pathogenic hypotheses

### The common pathogenic factors

Although the etiology of MDD is still unclear, it is widely accepted that MDD is associated with multiple pathogenic factor. In addition to well-known mental factors, MDD is also related to genetic factors, social stress, and even other common chronic diseases. Therefore, the etiology of MDD cannot be described from the perspective of a single factor.

#### Genetic factors

Although the etiology of MDD is still unclear, numerous studies have been performed and various models have been employed to explore the genetic factors, environmental factors and gene-environment interactions related to the disease.^24^ Recent family, twin, and adoption studies suggests that genetic factors play a crucial role in the occurrence of MDD.^25^ As a genetically diverse illness, MDD has a heritability of 30–50%.^26^ Over 100 gene loci, including those associated with presynaptic vesicle trafficking (PCLO), dopaminergic neurotransmission (a primary target of antipsychotics), glutamate ionotropic receptor kainate type subunit 5 (GRIK5), and metabotropic glutamate receptor 5 (GRM5), and neuronal calcium signaling such as calcium voltage-gated channel subunit alpha1 E (CACNA1E) and calcium voltage-gated channel auxiliary subunit alpha2 delta1 (CACNA2D1), are found to be associated with an increased risk of MDD by genome-wide association studies.^19,27,28^ In addition, rare copy number variants are also identified to be related to MDD risk, there may be three copy number variants (CNV) loci associated with Prader-Willis syndrome: 1q21.1 duplication, 15q11-13, and 16p11.2. However, no single genetic variation has been found to increase the risk of MDD thus far.^26^ Genome Wide Association Studies (GWAS) identified 178 genetic risk loci and proposed over 200 candidate gene, using of biobank data, novel imputation methods, combined with clinical cases improved the ability to identify MDD specific pathways.^29^ In the study of human MDD transcriptome, there are defects in the transcription levels of somatostatin (SST) in the subgenus anterior cingulate cortex and amygdala of MDD patients,^30,31^ and SST levels are directly involved in the cellular processes that affect the synaptic output of intermediate neuronal circuits.^32^ Recent studies revealed that gender specific genomic differences in MDD patients, the downregulation of the MDD-related gene Dusp6 in females leads to an increased susceptibility to stress, but this expression is not present in male mice.^33^ In addition, studies of drug gene interactions, transcriptional genes associated with the risk of MDD are also reported, such as D2 subtype of the dopamine receptor (DRD2) and fatty acid desaturase (FADS),^34^ which may serve as promising new targets for therapeutic intervention points. Thus, genetic variants are expected to have only minor effects on the overall risk of disease, and various hereditary factors combined with environmental factors such as stress are likely more essential for the development of MDD.^35^

#### Stress factors

In addition to heritable factors, environmental influences such as stress also significantly contribute to the development of MDD, both independently and in conjunction with genetic factors.^26^ Numerous studies have suggested that adverse life events can lead to the development of MDD.^18^ A major depressive episode always follows a traumatic or stressful life event. In particular, severe events such as job loss, extramarital affairs and divorce are known to provoke the onset of the disease.^36^ The exact pathological mechanism by which social stress results in the development of MDD is still not known, mainly due to the difficulty of separating social factors from genetic factors in patients and the impracticality of exposing disease model animals to relevant environmental factors. It has been proved that the changes in the structure and function of neurons may occur under the chronic stress and lead to the occurrence of MDD.^37,38^ In some MDD patients, stress leads to long-term elevated glucocorticoids, resulting in synaptic structural changes and remodeling, and the stress-induced hyperactivity of the HPA axis leads to negative feedback imbalance of the HPA axis, which is also related to depression.^39^ Studies on damage to microglia and astrocytes suggest the significance of glial cells in the development of environmental factor-induced depression-like behaviors in mice.^40^ In addition, our previous studies proved that chronic environmental stress-induced depressive-like behaviors in mice can be dependent on purinergic ligand-gated ion channel 7 receptor (P2X_7_R) activation in astrocytes.^41^

#### Comorbidity factors

The existence of various physiological and psychological comorbidities in patients with depression reveals a clear link between physical and mental health, which has given us a better understanding of MDD. The presence of MDD is a risk factor for a variety of complications, including neurodegenerative diseases (such as dementia, Alzheimer’s disease, and Parkinson’s disease), cardiovascular diseases (such as ischemic coronary artery disease and myocardial infarction), metabolic and endocrine diseases (such as obesity in females and diabetes in males), and some autoimmune diseases.^42,43^ The relationship between the onset of MDD and several diseases is complex and potentially bidirectional in nature.^44^ The impact of depression on society and the economy is increased by the existence of comorbidities.^45^ Specifically, in 2018, comorbid disorders rather than MDD itself were responsible for 63% of all costs related to MDD in the United States.^46,47^ Furthermore, compared to people without depression, patients with MDD have been demonstrated to have a shorter life expectancy.^48^ Additionally, the worsening of comorbidities could be a factor in the premature mortality of MDD patients.^44^

### Neurotransmitter and receptor hypothesis

The traditional monoamine theory contends that in addition to common pathogenic factors, deficiencies in monoamine neurotransmitters, such as serotonin (5-HT), dopamine (DA) and norepinephrine (NE), are the root cause of clinical depression.^49^ Selective serotonin reuptake inhibitors (SSRIs), a class of antidepressants that have been proven to successfully treat clinical depression, were developed in response to this hypothesis, which was derived primarily on the basis of the pharmacological mechanism of drug that were accidentally discovered to act as antidepressants. It is also crucial to note that astrocytes express NE transporter (NETT) and 5-HT transporter (SERT), which are the targets of some traditional antidepressants.^50^ A previous study suggested that the function of astrocytes can be directly regulated by SSRIs.^51^ Monoamine oxidase (MAO) activates the metabolism of adrenaline and triggers calcium signaling in astrocytes,^52^ which suggests that antidepressants may directly affect astrocytes by preventing them from reabsorbing monoamines.

#### Serotonin (5-HT)

An essential neuromodulatory transmitter with specific neuroplastic properties is serotonin. Numerous investigations have demonstrated that 5-HT is intimately related to the pathophysiological process of major depression. The 5-HT hypothesis primarily asserts that a decrease in the 5-HT level is a risk factor for depression.^53^ In addition, low levels of 5-HT and L-tryptophan, which is a precursor of 5-HT,^54^ in blood platelets are also found in depressed people. Additionally, long-term treatment with fluoxetine, a typical SSRIs, reverses the stress-induced reduction in the quantity of astrocytic cells in the hippocampus in a tree shrew model of depression.^55^

5-HT receptors, which are mostly found on the bodies and dendrites of neurons, play a role in the pathogenesis of MDD.^56^ To date, 5-HT receptor subfamilies comprising 14 different receptor subunits expressed in various brain regions, namely, 5-HT_1A_, 5-HT_1B_, 5-HT_1D_, 5-HT_1E_, 5-HT_1F_, 5-HT_2A_, 5-HT_2B_, 5-HT_2C_, 5-HT_3_, 5-HT_4_, 5-HT_5A_, 5-HT_5B_, 5-HT_6_ and 5-HT_7_, have been reported. Among these 5-HT receptor subtypes, the 5-HT_1_, 5-HT_2_, 5-HT_6_, and 5-HT_7_ subtypes are expressed on brain and spinal astrocytes in humans and rodents. Numerous 5-HT receptors expressed on astrocytes are G-coupled proteins that are associated with changes in the concentration of free cytosolic calcium ([Ca^2+^]_i_). These changes may trigger the release of a variety of astrocyte-derived signaling modulators, which may control neuronal activity.^57^ In astrocytes, 5-HT has a strong effect on the 5-HT_2B_ receptor.^58^ 5-HT receptors have been extensively studied to determine the pharmacological mechanism of antidepressants, and many novel pharmaceutical preparations are being investigated. For example, some novel antidepressants function as agonists of the 5-HT_1A_, 5-HT_2B_, or 5-HT_4_ receptor or antagonists of the 5-HT_1B_, 5-HT_2A_, 5-HT_2C_, 5-HT_3_, 5-HT_6_, or 5-HT_7_ receptor.^59^

**Fig. 2:**
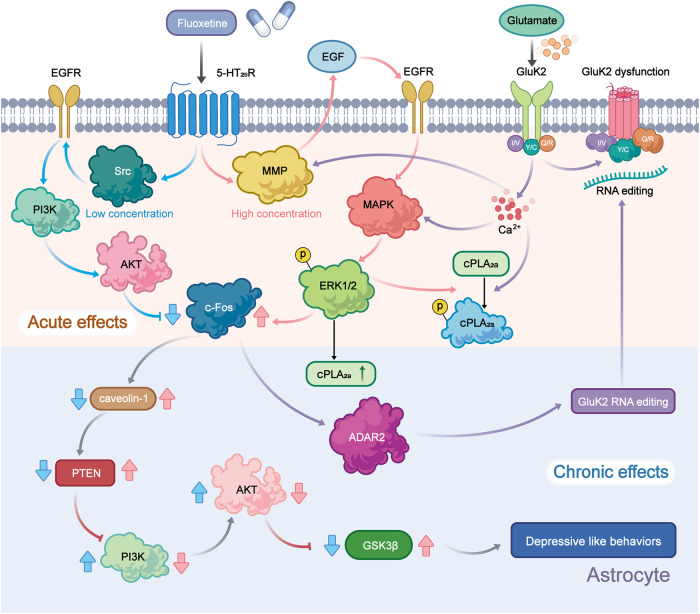
Schematic illustration of the pharmacological mechanism of fluoxetine in astrocytes. Acute treatment with fluoxetine at low concentrations (green arrows) stimulates Src, which phosphorylates EGF receptors by activating 5-HT_2B_ receptors (5-HT_2B_R) and activates the PI3K/AKT signaling pathway. AKT phosphorylation induced by fluoxetine at low concentrations inhibits the expression of cFos and subsequently decreases the expression of caveolin-1 expression (chronic effects), which in turn decreases the membrane content of PTEN, induces phosphorylation and stimulation of PI3K and increases the phosphorylation of GSK3β, thus suppressing its activity. At higher concentrations, fluoxetine (red arrows) stimulates metalloproteinases (MMP) by activating 5-HT_2B_R and induces the release of growth factors, which stimulates EGF receptors and activates the mitogen-activated protein kinases (MAPK)/ERK_1/2_ signaling pathway. ERK_1/2_ phosphorylation induced by fluoxetine at high concentrations stimulates the expression of cFos and subsequently increases the expression of caveolin-1 (chronic effects), which inhibits PTEN/PI3K/AKT/GSK3β,^60^ ultimately leading to MDD like behavior. At high concentration, fluoxetine can also stimulate the activation of cPLA_2a_ by the transactivation of EGFR/MAPK/ERK_1/2_ pathway, and the activated ERK_1/2_ can also increases the expression of cPLA_2a_ at chronic treatments.^61^ In addition, the increased expression of cFos induced by fluoxetine can further increases the RNA editing of GluK2 by increasing the expression of ADAR2 at the chronic treatments, the function of the edited GluK2 by fluoxetine is down-regulated, which causes the acute glutamated induced Ca^2+^-dependent ERK phosphorylation is suppressed.^63^ Adobe Illustrator was used to generate this figure

Administration of fluoxetine in different concentrations to astrocytes expressing the 5-HT_2B_ receptor may activate distinct signaling pathways to control gene expression. Fluoxetine reduces the mRNA expression of c-Fos through the PI3K/AKT signaling pathway after acute application at concentrations below 1 μM, while the treatments with the higher doses (above 5 μM), it increases the gene expression of c-Fos via the MAPK/ERK signaling pathway in astrocytes.^60^ Then, in the nucleus, the altered transcription factor c-Fos can further biphasic change the expression of caveoline under the chronic treatments, thus the alteration levels of caveoline on cellular membrane can finally affect the downstream activation of PTEN/PI3K/AKT/GSK3β^60^. The GSK3β polymorphisms are associated with the high risk of MDD in Chinese Han Population.^61^ In our recent reports, the activation of GSK3β is also increased in the sorted astrocytes from the MDD-related stress-treated mice model and MDD clinic patients’ plasma.^62^ In addition, after fluoxetine-mediated stimulation of the 5-HT_2B_ receptor in astrocytes, epidermal growth factor receptor (EGFR) is transactivated and subsequently activates the MAPK/ERK and PI3K/AKT signaling cascades, which control the expression of mRNA or proteins that may be linked to mood disorders, such as SERT. Ca^2+^-dependent phospholipase A2 (cPLA_2_), adenosine deaminase acting on RNA 2 (ADAR2), and kainate receptor subtype 2 (GluK2) are all involved in kainate receptor signaling.^63,64^ These discoveries promise astrocytic 5-HT_2B_ receptors can be the potential pharmacological target of SSRIs (Fig. 2).

#### Norepinephrine (NE)

NE released by the locus coeruleus (LC) can participate in regulating various neural functions, such as smell, movement, and sensation.^65^ It is significant to note that after being released, noradrenaline (NA) is not restricted to the area around the synaptic cleft and can reach nearby glial cells.^66^ Atomoxetine is a norepinephrine reuptake inhibitor (NRI) clinically used for the treatment of MDD. After systemic inflammatory attack with bacterial lipopolysaccharide (LPS), atomoxetine can decrease neuroinflammation in the rat cerebral cortex.^67^

The bioavailability of 5-HT and NE are increased by antidepressants called serotonin/norepinephrine reuptake inhibitors (SNRIs), which belong to antidepressants. Currently, new SNRIs, including duloxetine (DXT),^68^ desvenlafaxine (DVS),^69^ and venlafaxine,^70^ are widely used in MDD patients resistant to other treatments. Chronic treatment with DXT increases the expression of connexin 43 (Cx43), a crucial component of astrocyte gap junctions, in the rat PFC, preventing chronic unpredictable stress-induced dysfunction of astrocyte gap junctions and reversing the depressive-like behaviors caused by gap junction inhibition.^71^ A novel therapeutic target for MDD is transforming growth factor β1 (TGF-β1), the expression of which is controlled by antidepressants. Venlafaxine has also been found to exert neuroprotection by boosting the production of type 2 fibroblast growth factor (FGF-2) and transforming growth factor 1 TGF-β1 in astrocytes following stroke.^72^ However, the expression of protein markers of astrocytes and neurons is unaffected by DVS, and the chronic unpredictable mild stress (CUMS)-induced reduction in the levels of myelin- and oligodendrocyte-related proteins can be prevented by DVS.^69^ DVS may reduce oligodendrocyte dysfunction in the CUMS mouse model by altering cholesterol production and reducing depression-like phenotypes.^69^

#### Dopamine (DA)

There is increasing evidence that people with depression have reduced dopamine neurotransmission.^73^ Astrocytes in the lateral habeula are involved in regulating depressive-like behavior,^74^ whereas the reward circuit is mediated by the striatum.^75^ The dorsolateral part of the striatum is linked to the drug-seeking behavior and drug addiction associated with psychiatric disorders. As the major input to the basal ganglia, the striatum and related nuclei are linked to psychiatric morbidity, while the chronic stress reduces dopamine levels in areas such as the striatum and hippocampus.^76^ Due to processes involving dopamine D2 receptor signaling,^77^ the glutamine level increases in the presence of dopaminergic lesions and decreases in the presence of a high DA level.^78^ DA signaling is considered to play a key role in astrocyte-neuron crosstalk in the striatum.^79^ Sulpiride is an antidepressant that blocks the ability of the GLT-1 inhibitor TFB-TBOA to induce synaptic depression^80^ and partly attenuates the impact of fluorocitrate (a metabolic uncoupler that blocks aconitase in the tricarboxylic acid (TCA) cycle) on synaptic output. According to these results, astrocyte dysfunction results in an increase in DA levels, which decreases neuronal activity resulting from the binding of DA to dopamine D2 receptors,^80^ which generates neuronal depolarization, reducing DA selectivity at dopamine D1-like receptors and promoting DA inhibition through dopamine D2 receptors, which may contribute to increasing extracellular glutamate levels.^81^ An increase in DA signaling brought on by compromised astrocyte activity may induce a long-lasting change in striatal neurotransmission^80^ since DA signaling is crucial for both structural and synaptic plasticity.^82^

#### Glutamate

Glutamate is the main excitatory neurotransmitter in the central nervous system (CNS)^83^ and can be released by neurons through exocytosis, which in turn activates extracellular N-methyl-D-aspartate receptors (eNMDARs) in neurons, leading to synaptic loss.^84^ Exosynaptic glutamate also contributes to metabolism in neurons and astrocytes. When exosynaptic glutamate is taken up by astrocytes, it can become a substrate for glutamine synthesis or be metabolized by astrocytes and neurons.^85^ In addition, extracellular glutamate can also promote glucose uptake by astrocytes and inhibit glucose uptake by neurons. Therefore, glutamate is an important signal that mediates the interaction between central neurons and astrocytes, and its normal release and transport are the result of the functional cooperation between neurons and astrocytes. Glutamate homeostasis and neurotransmission play a major role in the onset of depression and anxiety. Studies have shown that glutamate levels in frontal cortex samples from autopsied patients with severe depression are increased, and antidepressants can restore normal glutamate levels.^86^ It has been observed in animal models that sustained glucocorticoid stimulation can increase the excitability of glutamatergic neurons and simultaneously decrease the number and plasticity of astrocytes, in addition to decreasing neuronal dendrite connectivity in the hippocampus and frontal cortex, leading to depression.^87^

It is well-documented that astrocytes have a wide range of modulatory functions that may either increase or decrease the release of many different neurotransmitters. Specifically, astrocytes are essential regulators of glutamatergic neurotransmission, and reuptake of glutamate by astrocytes regulates excitatory synaptic activity.^85^ When a large amount of glutamate is released from neuronal vesicles, glutamate clearance is mainly achieved by glutamate transporters (EAATs) on the membrane of astrocytes, which transport excess glutamate into astrocytes, where it is converted to glutamylamine through the action of glutamine synthase, reducing damage to neurons.^88,89^ In the classic glutamate-glutamine cycle, astrocytes and neurons convert glutamate to the nonexcitatory amino acid glutamine, which is then released back into the extracellular space and absorbed by neurons. Alterations in astrocytic glutamate clearance are known to occur in schizophrenia and other psychiatric illnesses, and mice with glutamate/aspartate transporter (GLAST) deletion show phenotypic abnormalities such as mental and behavioral deficits.^90,91^

#### Adenosine triphosphate (ATP)

Ectonucleotidases that are found in synapses can catabolize extracellular ATP to produce adenosine, and synapses also contain bidirectional nucleoside transporters that can release adenosine.^92^ Adenosine primarily stimulates inhibitory A1 and facilitatory adenosine receptors (A_2A_R) to play function.^93^ Notably, depressive behavior is linked to purinergic signaling. Depressive-like symptoms are exacerbated by activation of P2X_7_R in glial cells.^94^ Polimorphisms at P2X_7_R increase vulnerability to mood disorders whereas P2X_2_R-mediated neuronal activity is decreased in mice exposed to chronic stress due to insufficient ATP release from astrocytes.^95^ According to our earlier studies, chronic sleep deprivation (SD) can cause depressive-like behaviors by increasing extracellular ATP levels in vivo.^41^ Acting through P2X_7_R and FoxO3a cascade ATP inhibits expression of the 5-HT_2B_ receptor, the decrease in extracellular ATP levels caused by chronic stress and an increase in ATP levels caused by SD are both linked to depressive-like behaviors.^41^ In detail, the elevated extracellular ATP induced by SD stress stimulates P2×_7_R and down-regulates the expression of 5-HT_2B_R by suppressing the activation of AKT, which inhibits the phosphorylation of FoxO3a and promotes its transportation into the nucleus, the reduced 5-HT_2B_R alleviates the inhibition of STAT3 to cPLA_2_, the activated cPLA_2_ further increases the release of AA and PGE2, these indicators have high relationship with the depressive-like behaviors, because in P2X_7_R knockout mice, the above changes of these indicators and behavioral performance are all eliminated.^41^ This increased activation of cPLA_2_ and the elevated levels of AA and PGE2 in astrocytes are supported by our discoveries in MDD patients’ plasma.^62^

After building a stress injury model in rats through maternal separation (MS), it is found that MS obviously reduces the total length of apical dendrites, however, the use of A_2A_R antagonists could prevent synaptic loss^96^ and reverse behavioral, electrophysiological, and morphological damage caused by MS,^97^ this is related to the activity reconstruction of the HPA axis. In another study, the abnormally increased A_2A_R in the lateral septum(LS) is a key factor in recurrent stress for leading to depressive-like behaviors. This function is mainly achieved by the increased activity of A_2A_R-positive neurons and the inhibited activity of ambient neurons, associating with the neural circuits of dorsomedial hypothalamus(DMH) and lateral habenular(LHb).^98^

Caffeine is an adenosine receptor antagonist, and epidemiological studies have shown that the intake of caffeine is closely related to the occurrence of suicide^99^ and depression.^100^ Since A_2A_R polymorphisms are associated with emotional problems, adenosine A_2A_R overexpression leads to emotional dysfunction, and A_2A_R blockade protects against the persistent emotional disturbance brought on by stress.^101^ Moreover, animal experiments have demonstrated that A_2A_R are upregulated in chronic stress animal models.^102^ Additionally, neuronal A1 receptors exhibit hypofunction caused by a decrease in astrocyte-derived adenosine levels;^103^ this decrease, as well as depressive-like behavior, can be reversed by certain antidepressants.^104,105^

### HPA axis hypothesis

Stress and MDD are closely related, and stressful life events can often lead to depressive episodes. The activation of the HPA axis by stress can cause cognitive and emotional changes.^106^ An increase in HPA activity is one of the most common neurobiological alterations in depressed people. Studies have shown that the main factor contributing to the elevation of hypothalamic-pituitary activity is the increased production of corticotropin-releasing hormone (CRH). In addition, pituitary adrenal corticotropic hormone (ACTH) is released in response to CRH, which in turn triggers the adrenal cortex to release glucocorticoids (GCs).

#### Glucocorticoids

The HPA axis, a component of the neuroendocrine system, is commonly associated with the stress response. Hyperactivity of the HPA axis is thought to be an important pathophysiological mechanism underlying depression. High HPA activity is among the most typical neurobiological alterations in depressed individuals. The HPA axis is the primary stress response system that produces GCs, which are a class of steroid hormones. There is evidence that GCs, which are released in response to stress, are harmful to neurons in various brain regions. The hypothalamic paraventricular nucleus (PVN) rapidly secretes CRH and arginine vasopressin (AVP)^107^ when the HPA axis is activated by stress. The anterior pituitary is stimulated by CRH and AVP to produce ACTH, which in turn increases the release of GCs into the bloodstream.^108^

The GC and mineralocorticoid (MC) receptors GR and MR are members of the nuclear receptor (NR) superfamily. Both NRs can be triggered by binding to either MCs (such as aldosterone) or GCs (such as cortisol). However, the affinity of MR for its ligands is 10 times higher than that of GR for its ligands.^109,110^ GRs are expressesd at higher levels and particularly concentrated in the pituitary and hypothalamus, as well as a variety of regions of the limbic system (including the amygdala, hippocampus, and PFC), which are important for cognitive and psychological functions.

To prevent loss of control over the HPA axis, GCs exert negative feedback on the axis in all regions involved (the limbic system, hypothalamus, and pituitary). Some data suggest that HPA axis imbalance and high levels of GCs play a core role in the pathogenesis of MDD and suggest that GR may serve as an important target for treating depression.^111^

#### Thyroid hormone

Thyroxine (T4) and triiodothyronine (T3) are the two primary Thyroid hormones (THs) that regulate metabolism, protein synthesis, the growth of bones, and nervous system development. Thyrotropin-releasing hormone (TRH), which regulates the synthesis of thyroid-stimulating hormone (TSH) by the anterior pituitary gland, is mostly produced by neurons in the PVN. TSH stimulates the thyroid gland to produce T3 and T4. The levels of serum-free T4 and free T3 are regulated by negative feedback from pituitary TSH release. Tissue deiodinase mostly transforms T4 into the less physiologically active metabolite reverse T3 and the more biologically active metabolite T4.^112^

Overactivity of the HPA axis may be caused by damaged astrocytes and aberrant GR function. The HPA and hypothalamic-pituitary-thyroid (HPT) axes are inextricably linked. The most important related finding is that cortisol directly affects TRH secretion (which regulates TSH release), potentially through the response of GCs to TRH mRNA expression in neurons. According to research, hypercortisolemia may result in a reduction in TRH mRNA levels in the mid-caudal PVN.^113^ TRH expression in the PVN is lower in nonpsychiatric patients treated with corticosteroids, and the mRNA levels of TRH are lower in the PVN of depressed patients who have recurrent suicidal thoughts. This suggests that the effect of hypothalamic TRH is weaker in these individuals.

THs are required for neuronal growth and function not only in the periphery but also in the CNS,^114^ where they promote the formation of microglia, astrocytes, including radial glial cells, and oligodendrocytes. The role of THs in glial cells is becoming clear because of new discoveries in the field of glial cell biology. THs affect the shape and proliferation of astrocytes, as well as the organization and expression of GFAP/vimentin, and boost GS activity.^115^ T3 has an effect on glial morphology and hence on glial function in the adult brain; therefore, it also has an effect on neuron-glia interactions.^115,116^ It has been shown that T3 induces astrocyte proliferation by autocrine production of growth factors such as epidermal growth factor (EGF) and FGF-2. Apart from their proliferation-promoting impact, these growth factors increase and modify the pattern of deposition of the extracellular matrix components laminin and fibronectin, therefore boosting cell adherence and attachment to the substratum. Together with the discovery that animals with hypothyroidism and mice with TH receptor mutations display significant defects in glial development, these findings indicate that astrocytes are TH targets and that TH can protect neurons and astrocytes from glutamate toxicity.^115^

#### Estrogens

The hippocampus is closely related to memory and learning, and estrogen plays an important role in these processes. Estrogen increases the proliferation, migration, and differentiation of neurons in the dentate gyrus to maintain hippocampal function and is also important for controlling the HPA axis.^117^

Estrone (E1), estradiol (E2), and estriol (E3) are the three physiological estrogens; among these estrogens, E2 is the most active, and its level quickly decreases throughout menopause.^118^ E2 has been demonstrated in numerous studies to alter systems involved in the pathophysiology of depression, including the serotonin and norepinephrine systems, and to considerably alleviate depressive symptoms in animal models. Estrogen therapy can decrease the quantity of 5-HT_1_ and β-adrenergic receptors while increasing the quantity of 5-HT_1_ receptors.^119^ In addition, estradiol may influence the pathogenesis of male MDD patients.^120^ In animal models, E2 has been shown to alleviate depressive-like behavior.^121,122^ Estrogen receptor 1 (ER1) and estrogen receptor 2 (ER2) are transcription factors that are members of the NR family. Activating ER2 with a range of ER2 agonists has been reported to reduce stress-induced HPA activity and anxiety-like behaviors.^123,124^

Astrocytes are estrogen targets,^125^ as both ER1 and ER2 receptors are present on the astrocyte membrane or intracellularly in astrocytes. The transmembrane receptors ER and GPR30 have been shown to facilitate nongenomic and fast estrogen signaling in astrocytes, contributing to the neuroprotective effects of E2. In mature astrocytes differentiated from human induced pluripotent stem cells (iPSC)-derived astrocyte progenitors, ketamine can exert rapid antidepressant effects through the activation of amino-3-hydroxy-5-methyl-4-isoxazole propionic acid (AMPA) glutamate receptors, and estrogen enhances this effect of ketamine by increasing the gene expression of AMPA receptor subunits.^126^

#### Leptin

The obese gene (OB) encodes the hormone leptin, which is derived from adipocytes and the stomach and exerts its function through a specific receptor (OB-R). Leptin controls the function of the HPA axis^127^ via its receptor in the hypothalamus. The cerebral cortex, hippocampus, hypothalamus, dorsal raphe (DR) nucleus, arcuate nucleus, and solitary tract nucleus are some regions of the brain that can express leptin receptors. Increasing experimental data have recently shown that leptin is linked to the pathological and physiological processes of numerous mental illnesses and plays a vital regulatory role in the CNS.^128,129^ According to our previous reports, leptin can enhance the pharmacological effects of fluoxetine in astrocytes sorting from GFAP-GFP transgenic mice.^130^ Leptin selectively increases the expression of the astrocytic 5-HT_2B_ receptor by activating the JAK2/STAT3 pathway, and fluoxetine in turn stimulates the 5-HT_2B_ receptor and increases the secretion of brain-derived neurotrophic factor (BDNF) from astrocytes in vivo, thus ameliorating depressive-like behaviors.^130^ All of these findings indicate leptin’s potential to boost protein expression and functionally stimulate SERT.

### Cytokine hypothesis

MDD is accompanied by changes in the levels of proinflammatory cytokines and trophic factors, including BDNF, interleukins (IL-1β, IL-6), and tumor necrosis factor alpha (TNF-α). Increasing data suggest that the production of certain cytokines by brain astrocytes plays a significant role in the pathogenesis of MDD.

#### Oxidative stress

Oxidative stress (OS), which is caused by an imbalance between antioxidants and reactive oxygen species (ROS), can harm proteins, lipids, or DNA. The activity of monoamine oxidase, the enzymes that break down monoamines such as DA, 5-HT and NE, is influenced by ROS and in turn can increase ROS production in mitochondria. The brain is more vulnerable to OS than other organs. In depression, OS plays a crucial role.^131,132^ The brain is particularly sensitive to OS due to numerous variables, including rapid oxidative energy metabolism (a process through which ROS, which are harmful molecules, are constantly produced), high levels of unsaturated fatty acids (which are vulnerable to lipid peroxidation), and relatively low intrinsic antioxidant capability.^133^ Adults with MDD exhibit ROS-mediated reductions in nitric oxide (NO)-dependent dilation.^134^

Thioredoxin reductase, heme-oxygenase 1, glutathione, and glutathione peroxidase are only a few of the ROS-detoxifying enzymes that are abundant in astrocytes.^135^ Astrocytes are the major producers of glutathione in the brain because they express a system xc-cyttine/glutamate antiporter, which does not exist in neurons; hence, neurons cannot synthesize glutathione. Notably, astrocytes can protect nearby neurons against toxic dosages of NO, H_2_O_2_, and superoxide anion in combination with NO, iron, or 6-hydroxydopamine in coculture systems,^135^ indicating that neurons rely on the strong antioxidant capacity of astrocytes for protection against OS. Nuclear factor erythroid 2 (Nrf2), a redox-sensitive transcription factor required for coordinating the cellular antioxidant response, can be activated by astrocytes. In our recent study, lithium salt (Li^+^) was found to effectively alleviate ischemia-induced anhedonia in mice by suppressing the production of mitochondrial ROS in glial cells.^136^

Recent investigations have indicated that MDD is caused by increased ROS production and promotes inflammation.^137^ The brain has weak antioxidative defenses and a high oxygen consumption rate, making it particularly susceptible to OS. Inflammasomes in microglia can be activated by ROS, which causes inflammatory cytokines, including TNF-α, IL-1β, and IFN-γ, to be produced.^138^ Neuroendocrine-immune activities can be compromised by inflammation, which can also result in numerous disorders, such as MDD. Proinflammatory cytokines have become pathological indicators of MDD, and using the right antioxidants to combat ROS may be a useful method for treating MDD.

#### Proinflammatory cytokines

Higher levels of inflammation increase the chance of developing new-onset depression.^138,139^ Although depression can cause inflammation, its cause is still unclear and may be influenced and regulated by immune cells, inflammatory cytokines, and the nervous system. In addition to contributing to the etiology of depression, activation of proinflammatory signaling pathways occurs as a result of elevated OS.^140^ Evidence suggests that MDD is associated with the immune response, as shown by increased levels of IL-1β, TNF-α, and IL-6.^141^ LPS-induced astrocyte activation also contributes to the symptoms of MDD. Systemic treatment with LPS induces depressive-like behaviors and increases the production of inducible nitric oxide synthase (iNOS), IL-1β, TNF-α, and GFAP in the hippocampus and cortex. Inhibition of activated astrocytes reduces neuroinflammation. These alterations are followed by amelioration of LPS-induced depressive-like behaviors.^142^

#### Neurotrophic factors

In the vast majority of patients with severe depression, antidepressants affect the levels of neurotrophic factors. For example, the primary regultaory factor of neuronal survival, growth, and differentiation during development is BDNF. For the treatment of depression, targeting signaling transduction by BDNF and its receptor, tropomycin receptor kinase B (TrkB), is essential.^143,144^ Recent research has shown a link between decreased hippocampal neurogenesis and low levels of BDNF and glial-derived neurotrophic factor (GDNF) in the brains of depressed individuals.^145^ Under normal conditions, astrocytes release various nutrients and cytokines. After cell reactivation, the secretion of these factors is further increased.^146^ According to previous studies, fluoxetine stimulates c-Fos expression and ERK_1/2_ phosphorylation, which in turn promotes BDNF production in astrocytes sorting from GFAP-GFP transgenic mice.^147^ Imipramine acts as an antidepressant by increasing the mRNA expression of BDNF in astrocytes. Fluoxetine also induces BDNF expression by activating cAMP-response element binding protein(CREB) through the PKA and/or ERK pathways.^148^

BDNF is an essential molecule for neural plasticity and development and is related to several CNS diseases. Currently, it is known that BDNF can regulate the activity of neurons and that it is produced not only by neurons but also by astrocytes.^149^ SSRIs and tricyclic antidepressants increase BDNF expression in cultured primary astrocytes, and BDNF overexpression in mouse hippocampal astrocytes is sufficient to promote neurogenesis and causes anxiolytic behavior.^149^ By promoting neurotransmitter release, facilitating vesicle docking, and upregulating the expression of synaptic vesicle proteins, BDNF, which is released by astrocytes in response to long-term antidepressant therapy, may assist in increasing synaptic plasticity at presynaptic terminals.^150^ In addition, astrocyte-secreted BDNF can stimulate adult hippocampal neurogenesis and may contribute to synaptic and structural plasticity that underlies the long-lasting behavioral effects of antidepressants.^150^ Astrocytes can secrete numerous nerve growth factors. Vascular endothelial growth factor (VEGF) is a member of the vasoactive growth factor family. It exerts its unique molecular effects by binding and activating endothelial cell tyrosine kinase receptors. VEGF is traditionally associated with angiogenesis and its stimulation. Recent evidence indicates, however, that it also influences nerve cells and plays a crucial role in hippocampal neurogenesis and neuroprotection.^151^

#### Inflammasomes

Neuroinflammation is a central pathophysiological mechanism and defining characteristic of MDD. Numerous elements in the periphery and CNS interact to generate neuroinflammation, thereby stimulating astrocytes. The nucleotide-binding domain and leucine-rich repeat protein-3 (NLRP3) inflammasome is one of the largest typical inflammasomes discovered thus far. It is composed of pro-Casp-1 protein, NLRP3, and apoptosis-associated speck-like protein (ASC).^152^ The sensitization of the NLRP3 inflammasome and the suppression of BDNF synthesis result in MDD.^153^ In our research, SD is found to reduce BDNF levels and induce depressive-like behaviors in the sorted astrocytes from GFAP-GFP transgenic mice by activating the NLRP3 inflammasome.^130^ NLRP3 inflammasome activation causes astrocytes to produce more IL-1β and IL-18.^154,155^

The release of proinflammatory cytokines is the primary consequence of the activation of caspase-1, a component of the NLRP3 inflammasome. In addition, it has been observed that stimulating NLRP3 inflammasome assembly can induce depression-like behaviors in rodents exposed to LPS or CUMS.^156,157^ Research on the effect of astrocyte-specific NLRP3 knockout suggests that the astrocytic NLRP3 inflammasome exerts a significant effect on astrocytic pyroptosis via the Casp-1/GSDMD pathway in depression.^156,157^ Therefore, efficient NLRP3 inflammasome inhibitors are novel therapeutic agents for MDD. As we previously reported, chronic SD can specifically activate the NLRP3 inflammasome and decrease the level of BDNF in astrocytes to ameliorate depressive-like behaviors. Fluoxetine can suppress the effects of SD on astrocytes by stimulating astrocytic 5-HT_2B_ receptors directly.^147^ Additionally, in the middle cerebral artery occlusion (MCAO) stroke model of mice, Li^+^ can significantly attenuate GSDMD-mediated glial pyroptosis by regulating the AKT/GSK3β/TCF4/β-catenin signaling pathway, in which, the activation of AKT induced by Li^+^ can also increase the phosphorylation of FoxO3a and promote the transportation of FoxO3a from nucleus into cytoplasm, the reduced FoxO3a in nucleus dissolves its competition with TCF4 in order to confirm more β-catenin/TCF4 complex. The increased latter complex further up-regulates the expression and activation of STAT3 in nucleus, the latter further inhibits the activation of the NLRP3 inflammasome by increase UCP2 which can decrease the production of ROS from mitochondrion.^136^ This neuroprotective mechanism of Li^+^ after ischemia-reperfusion injuries contributes to the improved depressive-like behaviors, besides of motor and cognitive capacities.^136^

**Fig. 3:**
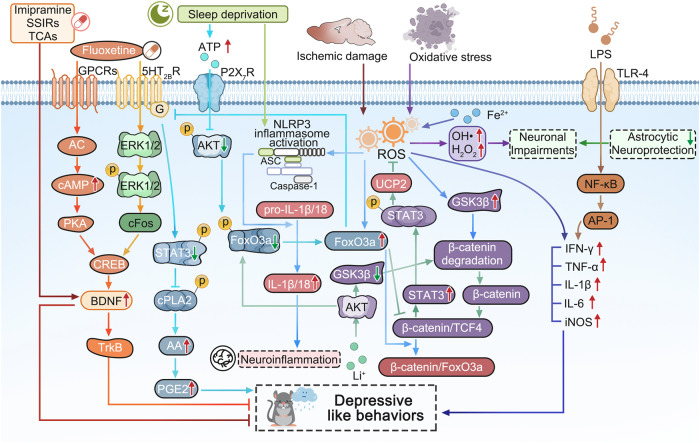
The molecular signaling schematic of cytokine hypothesis in the pathogenesis of MDD. The rodent performed the depressive like behaviors are impaired by some widely accepted risk factors, such as long-term sleep deprivation (SD), oxidative stress, lipopolysaccharide (LPS), ischemic damage and so on. Long-term SD can increase the extracellular ATP level, the latter inhibits the activation of AKT and the followed phosphorylation of FoxO3a by stimulating P2X7 receptors (P2X7R), the dephosphorylated FoxO3a translocates into the astrocytic nucleus, then the increased FoxO3a decreases the expression of 5-HT_2B_R expression, which results the reduced phosphorylation of STAT3 which increases the activation of cPLA2 and the followed release of arachidonic acid (AA) and prostaglandin E2 (PGE2), finally causing the depressive-like behaviors.^41^ Thus, antidepressant fluoxetine activates ERK_1/2_/cFos pathway by stimulating 5-HT_2B_R and AC/cAMP/PKA pathway by activating GPCRs in order to increase the activation of CREB and the level of BDNF and TrkB, which can alleviate the depressive like behaviors induced by long-term SD.^147,148^ As well as, imipramine, other SSIRs, and TCAs can also play antidepressive roles by increasing BDNF mRNA expression in astrocytes.^148^ Ischemic stroke can trigger the increase of reactive oxygen species (ROS) which can induce the activation of NLRP3 inflammasome and the release of IL-1β/18, resulting in the neuroinflammation, however, Li^+^ salt inhibits the activation of GSK3β and increases the phosphorylation of FoxO3a by activating AKT, which promotes the more FoxO3a transportation from nucleus into cytoplasm, and the reduced FoxO3a in nucleus lacks the competition with TCF4, the increased complex level of β-catenin and TCF4 further stimulates the expression and the phosphorylation of STAT3, which further induce the mRNA and protein expression of UCP2, then in mitochondrion, the increased UCP2 suppresses the production of ROS and results in the deactivation of NLRP3 inflammasomeincreases.^136^ Superoxidation of Fe^2+^ stimulates an increase in ROS, resulting in the production of inflammatory cytokines (including IFN-γ, TNF-α, IL-1β, IL-6) and inducible nitric oxide synthase (iNOS).^138^ While, the treatments of oxidative stress (OS) can produce a large number of ROS, such as OH• and H_2_O_2_, resulting in neuronal impairments, while astrocytes can play their neuroprotective role by antioxidation.^135^ Additionally, LPS can also increase TNF-α, IL-1β, and IL-6 by TLR-4/NFkB/AP-1 pathway and cause depressive-like behavior.^142^ Adobe Illustrator was used to generate this figure

In conclusion, there have been so many hypothesis to explain the pathogenesis of MDD associating with many booming researches (Fig. 3). However, it is still hard to adopt only one above hypothesis to completely reveal pathophysiology of MDD. The main problem may contribute to the limitations of the theoretical perspective and the limitations of detection methods. Some key scientific problems in the neurobiology of neurological and psychiatric disorders are still unclear, such as how to identify the pathological characteristic changes for mood disorders, how to metabolize the cerebral metabolic waste under the pathological condition,how to observe the instant interactions of neural cells and the real-time changes of intracellular organelles in the patients of MDD? In the pathological conditions, conducting research from the perspective of comprehensive collaboration of the whole body and increasing the proportion of new technological applications in research will open up the new paths to reveal the pathogenesis of MDD in the future.

## Interactions of multi-cells and multi-organs

Recently, increasing evidence has shown that pathological changes in a single cell type or brain region limited are insufficient explain the pathogenesis of MDD. This section mainly introduces the latest research on the pathogenesis of MDD, discussing the multiple interactions among neural cells and the multiple regulatory mechanisms between the brain and peripheral organs in detail.

### The interaction between neuron and glial cell

Over the past few decades, studies on MDD have identified decreased PFC activity and excitatory/inhibitory (E/I) imbalance as probable mechanisms underlying depression.^158^ Astrocytes are recognized to be essential for controlling neural network activity and to take part in higher brain activities.^159^ To explore efficient treatments for MDD, it is important to focus on how to regulate the E/I balance and neuronal remodeling.^160^

#### MDD-related marker proteins in neural cells

Astrocytes in the CNS form the neurovascular unit with neurons and blood vessels. The neurovascular unit mediates the exchange of nutrients and other functional substances between its components.^161^ The blood-brain barrier (BBB) consists of endothelial cells tight junctions, a continuous basement membrane and astrocytic end-feet. Two proteins expressed on astrocytes, connexin 30 (Cx30) and Cx43, have been linked to the pathogenesis of depression.^162^ Gap junctions that enable communication between astrocytes are formed by the membrane proteins Cx30 and Cx43.^163^ Chronic unpredictable stress (CUS) and acute stress both specifically reduce the expression of the gap junction-forming proteins Cx30 and Cx43,^164^ and the integrity of the BBB is weakened in mice lacking Cx30 and Cx43.^165^

In addition to being an essential component of the developing astrocyte cytoskeleton, GFAP serves as the main intermediate filament protein in adult astrocytes. Although increased expression of GFAP is commonly observed in reactive astrogliosis, postmortem results suggest that the frequency and intensity of reactive astrogliosis are decreased in the brains of patients with MDD.^166^ Accompanied by a decreased astrocyte density, the levels of GFAP and the GFAP intermediate filament domain are also reduced in brain samples from patients with MDD.^167^ Researchers have even proposed that the GFAP content in serum can be used to determine the severity of MDD,^168^ but this point is controversial.

AQP4, a kind of water channel, is mainly expressed on astrocytic end-feet in contact with blood vessels. The water channel AQP4 regulates the equilibrium of ions and water in the brain and is an essential part of the neurovascular unit. The vascular coverage of AQP4-immunopositive astrocytes in the orbitofrontal cortex (OFC) is lower in people with clinically significant depression than in psychiatrically healthy control patients.^169^ In another postmortem study, it was found that the coverage of blood vessels by AQP4-positive astrocyte terminals was reduced in the OFC of MDD patients.^170^ In addition, the K^+^-buffering capacity and presumably synaptic transmission are impaired in mice lacking AQP4, and impairment of these processes is associated with depressive-like behaviors.^171^ In our previous study, we reported that the expression of AQP4 was decreased by exposure to CUMS, which contributed to dysfunction of glymphatic circulation and depressive-like behaviors in mice.^172^ Additionally, the coverage of blood vessels by AQP4-positive astrocytic endfeet is decreased by 50% in MDD patients, indicating that decreased levels or mislocalization of AQP4 may contribute to the pathogenesis of MDD.^169,173^

S100B is produced and secreted by astrocytes in the gray matter,^174^ and changes in the levels of S100B in the blood and cerebrospinal fluid (CSF) of patients with MDD can cause glial cell dysfunction and damage.^175,176^ In individuals with MDD, the number of S100B-immunopositive astrocytes in the pyramidal layer of the bilateral hippocampal CA1 region is decreased.^177^ S100B secreted by damaged astrocytes can enter the extracellular space and CSF,^178^ and the level of S100B is increased in the dorsolateral prefrontal cortex (dlPFC) of patients with MDD.^179^ S100B levels are elevated in the CSF or serum of patients with MDD,^180^ which suggests that S100B is a potential diagnostic biomarker for depressive episodes associated with MDD.

Communication between neurons and microglia plays an important role in the pathogenesis of depression. C-X3-C Motif Chemokine Ligand 1 (CX3CL1)- C-X3-C Motif Chemokine Ligand 1 receptor (CX3CR1) and OX-2 membrane glycoprotein (CD200)-OX-2 membrane glycoprotein receptor (CD200R) form ligand-receptor pairs, and these molecules are the most important chemokines and clusters of differentiation in maintaining CNS homeostasis.^181^ CX3CL1 and CD200 are mainly expressed in neurons, and their receptors CX3CR1 and CD200R are expressed on microglia.^182^ Activated microglia and decreased expression of CX3CL1 in the hippocampus were observed in an LPS-induced depression model.^183^ CX3CR1-deficient mice show a temporary decrease in the number of microglia and a resulting deficiency of synaptic pruning, which may be related to neurodevelopmental and neuropsychiatric disorders.^184^ However, CX3CR1-deficient mice show significant resistance to stress-induced depressive-like behaviors.^185^ The level of CX3CL1 in the serum is increased in patients with moderate-severe depression compared with healthy subjects; thus, CX3CL1 could be used as a target for depression treatment.^186^ Patients diagnosed with MDD with comorbid cocaine addiction show higher serum levels of CX3CL1.^187^ Additionally, in a rat early-life social isolation (ESI) model, the expression of CD200 receptors in microglia is significantly reduced.^188^ Exposure to unavoidable tail shock causes a decrease in CD200R expression in the hippocampus and amygdala,^189^ and stress was also discovered to suppress CD200R expression in the hippocampus of rats.^190^

#### Synaptic plasticity

Long-term potentiation (LTP) serves as the physiological basis for learning and conditioned responses.^191^ Ketamine has a quick antidepressant effect, as it is a noncompetitive channel blocker of N-methyl-D-aspartate receptors (NMDARs).^192^ Excessive glutamate in the synaptic cleft activates synaptic metabotropic glutamate receptors (mGluRs), which lead to neural excitotoxicity.^193^ In a mouse model of chronic social defeat stress (CSDS), which causes depression, mGluR5 was shown to induce long-term depression (LTD). The major process responsible for synaptic plasticity is the mGluR-mediated LTD, which likely plays a significant role in the pathophysiological changes underlying depressive-like behaviors in the CSDS-induced depression paradigm.^194^

ATP can mediate the activity of the astrocyte-neuron network, and ATP is a signaling molecule that also controls synaptic plasticity.^195^ ATP can increase the expression of amino-3-hydroxy-5-methyl-4-isoxazole propionic acid receptors (AMPARs) by stimulating P2X_7_R and increasing the amplitude of miniature excitatory postsynaptic currents.^196^ Stress exposure is a major pathogenic factor in disease models and can increase Ca^2+^-dependent release of ATP from neurons, which causes excitotoxicity.^197,198^

Regulated in development and DNA damage response-1 (REDD1) is a stress response gene that can regulate development and the response to DNA damage. Virus-mediated overexpression of REDD1 in the rat PFC is sufficient to cause anxiety- and depressive-like behaviors and neuronal atrophy.^199^ According to postmortem studies, the volume of the dlPFC is smaller and the density of neurons in the dlPFC is lower in MDD.^200^ BDNF can modulate synaptic plasticity in the brain. TrkB is a functional receptor of BDNF.^201^ BDNF produces antidepressant-like effects by increasing synaptic plasticity in a mouse model of CUMS.^202^

#### Neuron-glia integrity

The term “tripartite synapse” was initially used to describe the intimate relationship between astrocytes and neurons at glutamatergic synapses, similar to the glutamate-glutamine cycle described above.^203^ Moreover, glutamic acid decarboxylase, an enzyme that transforms glutamate into γ-aminobutyric acid (GABA), also exists in inhibitory GABAergic neurons. Increased inhibitory neurotransmission, glutamatergic/GABAergic E/I imbalance, and chronic stress-related emotional dysfunction reduce PFC activity.^204,205^ In local circuits, various glutamatergic and GABAergic neurons interact in complicated ways to achieve E/I balance.^206^ A meta-regression analysis indicated that glutamine and glutamate levels are decreased in the PFC, which is correlated with the therapies to MDD.^207^ Global topological E/I imbalance in MDD is discovered through gene and protein expression of molecules related to inhibitory GABAergic and excitatory glutamatergic signaling in the postmortem MDD brains.^22,208,209^ It shows the imbalance in cortical-subcortical limbic regions with decreased GABAergic signaling and increased glutamatergic signaling.^210,211^ Meanwhile, GABAergic signaling is decreased in regions comprising the default mode network (DMN), while it is increased in the lateral prefrontal cortex (LPFC).^212,213^ Stimulating P2X_7_R in neocortical nerve terminals can block the reuptake of GABA and glutamate by the presynaptic membrane and promote the release of these two neurotransmitters in the cerebral cortex of rats and humans,^214,215^ and activation of P2X_7_R reduces the expression of GLAST.^216^ This results in neuronal damage, a reduced number of synapses, decreased neurogenesis, and even impairment of key cerebral circuits that regulate mood.

Astrocytes are fundamental elements in synapses, participate in synaptogenesis and maturation, and maintain synaptic homeostasis. Ionic homeostasis in the extracellular space is critical for central nervous system function.^217^ Astrocytes play an important role in maintaining extracellular K^+^ homeostasis in the CNS, as well as H^+^, Cl^-^, and Ca^2+^ homeostassis.^218^ In addition, it also plays an important role in maintaining transmitter homeostasis, in which glutamate and GABA play particularly important roles.^219^

In addition to the tripartite synapse, the more recent concepts of the four-part extracellular matrix and the microglial five-part synapse^220^ also support the idea that glial dysfunction plays key roles in the early pathological features common to psychiatric disorders.^221,222^ Under physiological conditions, microglia can play a neuroprotective role by producing cytokines. However, under pathological conditions, microglia can also affect the balance between excitatory and inhibitory synapses by phagocytosing synapses^223^ and activating inflammatory factors in microglia.^224^ In addition, the extracellular matrix (ECM) plays a significant role in maintaining normal communication in mature neural networks, which can limit the synaptic restriction of glutamate.^225^ The components of the ECM are mainly produced by neurons and astrocytes, and microglia can also regulate the remodeling of the ECM.^226^

### Interaction mechanism in multi-organs

Abnormalities in cytokine levels in the brain and peripheral organs, disruption of the brain/immune system balance, and dysfunction of communication between the peripheral organs and the brain can cause neuroinflammation and depressive symptoms. For instance, cirrhosis and depression have been linked to intestinal dysbiosis, which results in intestinal barrier disruption, increasing bacterial translocation. Increased bacterial translocation then activates circulating immune cells, which produce cytokines and induce systemic inflammation.^227^ In comparison with the healthy population, MDD patients have a much higher incidence and prevalence of chronic liver disease.^228^ Inflammatory bowel disease (IBD) and irritable bowel syndrome (IBS) with increased intestinal permeability, which may have both inflammatory and autoimmune sources, are common comorbidities of MDD and anxiety.^229,230^

#### Neuroendocrine-immune axis

Microglia secrete chemokines that disrupt the integrity of the BBB and increase the ability of immune cells to enter the brain parenchyma.^231^ The stress response is a complex array of behavioral, neuroendocrine, autonomic, and immunological responses that enable adaptation to unpleasant psychological and physiological stimuli.^232^ The HPA axis is a crucial endocrine system that orchestrates this response.^233^ Stress can activate microglia, which are considered important immunocytes of the CNS. Mediators released by activated microglia can stimulate the HPA axis and induce GC production.^39^ Similarly, high levels of GCs can also activate microglia, creating a vicious cycle.^234^

Tryptophan (TRP) can be converted into a variety of biologically active molecules, and more than 95% of TRP is metabolized to kynurenine (KYN) and its breakdown products, with only a small portion of TRP being converted to 5-HT.^235^ Indoleamine 2,3 dioxygenase (IDO) is an immune inducible enzyme that metabolizes TRP through the KYN pathway and plays an important role in the immune response.^236^ In the brain, KYN is metabolized to the neurotoxic substance quinolinic acid (QUIN).^237^

The primary GC in the HPA axis, corticosterone, plays a role in regulating the stress response in rodents. Stress, high GC levels, and serious depression are all linked. Analysis of transcriptomic changes associated with corticosterone-induced cytotoxicity revealed an association of neurite outgrowth-related genes with depression. Therapies for MDD may target the expression of genes involved in neurite formation, such as calpain 2 (Capn2), vesicle-associated membrane protein (Vamp7), and c-type natriuretic peptide (Cnp).^238^

Consumption of a high-fat diet (HFD; for approximately 16 weeks) results in anxiety and anhedonic behaviors, and 4 months of HFD consumption results in increased levels of corticosterone and blood glucose, which also activate the innate immune system, increasing the release of inflammatory cytokines (i.e., IL-6, IL-1β, TNF-α). The behavioral abnormalities that arise from long-term consumption of a HFD are quickly reversed by ketamine. Additionally, giving HFD-fed rats a P2X_7_R antagonist greatly alleviates their anxiety.^239^

#### Microbiota-gut–brain axis

In recent years, the microbiota-gut-brain axis has been reported to be disrupted in MDD. Stress stimulation can affect the gut microbiota, which in turn induces the production of inflammatory mediators (mainly IL-6 and IFN-γ) and a reduction in short-chain fatty acid levels.^240^ The increased level of inflammatory cytokines may be caused by disturbance of the gut microbiota, which may also disrupt the gut barrier.^241^ Alterations in the gut microbiota and inflammatory agents have an impact on the KYN pathway, metabolism, and toxin metabolism in the periphery.^242^ Proinflammatory cytokines or toxic byproducts resulting from microbiota alterations may pass through the BBB and enter the brain.^243^ This increases the levels of cytokines such as IL-1β and IL-6 and NLRP3 inflammasome activation in brain-resident cells.^244^ In particular, microglia and astrocytes are activated and undergo atrophy, respectively. These glial cell changes, which affect the brain networks involved in learning and memory, mood regulation, and emotional regulation, may cause depressive symptoms or anxiety episodes.^245^

According to clinical research, TRP and tryptophan catabolites (TRYCATs) may play a crucial role in psychiatric illnesses, including MDD. Peripheral and central inflammation can both stimulate the KYN pathway and trigger TRP metabolism and subsequent synthesis of various TRYCATs, including the toxic NMDAR activator QUIN,^246^ which influences glutamate transmission, has a variety of immunomodulatory effects and has both neurotoxic and neuroprotective effects on the CNS.^141^ Studies have proven that peripherally injected LPS increases the central and peripheral metabolism of TRP via the KYN pathway by exerting neurotoxic effects, inducing reactivation of microglia and astrocytes in the CNS.^247^ Excessive production of QUIN, an NMDAR agonist, stimulates the release of glutamate and inhibits reuptake, leading to neuronal excitotoxicity.^248^

#### Liver-brain axis

Patients with liver diseases often struggle with depression. According to one study on the frequency of liver disease and major depression in the United States, liver disease is linked to both major depression and suicidal thoughts.^249^ A further population-based cohort study discovered that patients with MDD had much higher prevalence and incidence rates of chronic liver disease than the general population.^228^ The incidence of depression is high in cirrhosis patients; moreover, depression is an independent predictor of mortality from cirrhosis.^250^

An internal metabolic mechanism regulated by the liver can control depressive-like behavior. A crucial enzyme in epoxyeicosatrienoic acid (EET) signaling in the liver is epoxide hydrolase (sEH). Chronic stress selectively exacerbates sEH-induced depression-related changes in the liver while dramatically lowering the plasma levels of 14,15-EET. Deletion of hepatic epoxide hydrolase 2 (Ephx2) (which encodes sEH) rescues the chronic mild stress (CMS)-induced decrease in 14,15-EET plasma levels.^251^ In a rat model of CUMS, electroacupuncture (EA) was found to downregulate P2X_7_R, NLRP3, and IL-1β expression in the prefrontal cortex and liver and relieved depression-like behavior.^252^

**Fig. 4:**
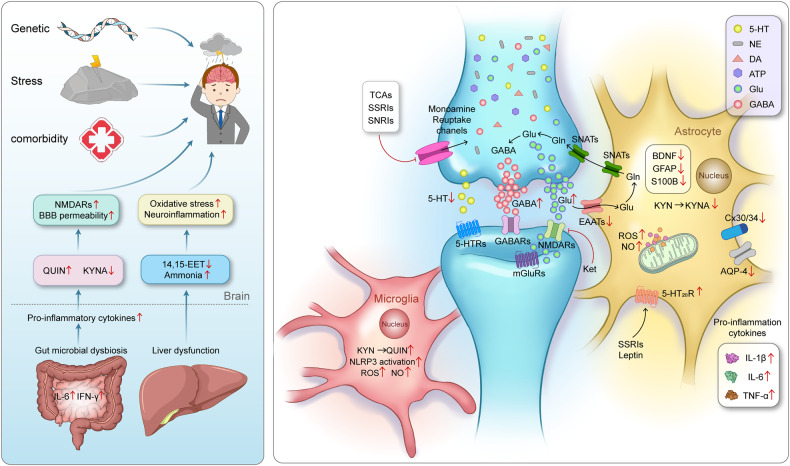
The pathogenesis of MDD is closely related to synapses, astrocytes, microglia, and their interactions as well as interactions among organ. Genetic factors, stress and comorbidities are considered the most common pathogenic factors of MDD^3^. The traditional monoamine theory contends that MDD may cause by the deficits in monoamine neurotransmitters.^49^ Moreover, the other abnormal increase of neurotransmitters in the synaptic cleft, such as glutamate, GABA and ATP, has the high relationship with the pathogenesis of MDD.^41,496^ The interaction between neurons and glial cells can induce the oxidative stress, pro-inflammatory cytokines released, the reduction of neurotrophic factors. The microbiota-gut-brain axis is clearly disrupted in MDD.^243,248^ When liver dysfunction occurs and causes OS and neuroinflammation in the brain, which also contribute to the pathophysiology of MDD.^497^ Adobe Illustrator was used to generate this figure

In summary, as shown in Fig. 4, although the etiology of MDD is still unclear, it is widely accepted that the common pathogenic factors of MDD are genetic, stress, and comorbidity.^3^ The levels of monoamine neurotransmitters (5-HT, NE, and DA) are insufficient in the synaptic cleft of MDD patients, correspondingly, the explored antidepressants such as tricyclic antidepressants(TCAs), SSRIs and SNRIs almostly act on the channels responsible for inhibiting reuptake of these neurotransmitters.^51^ Thus, according to these traditional pharmacological theories, these antidepressants always have the delayed clinical efficacy, this promises the potential new pharmacological mechanism still requires further study. As the well-known glutamate-glutamine cycle, astrocytes play key roles in resolving neuronal glutamate toxicity. However, under the MDD pathological condition, due to the decreased expression of EAATs in astrocytes, excessive glutamate in the synaptic cleft activates synaptic mGluRs, which leads to neuronal excitotoxicity.^194^ In addition, the overdose glutamate can also be decarboxylated by glutamate decarboxylase (GAD) to GABA and activates the GABA receptors on the postsynaptic membranes.^206^ In our previous studies, the expression of 5-HT_2B_ is selectively decreased in the sorting astrocytes from MDD model mice.^64^ The antidepressants SSRIs and leptin can increase the expression of the astrocytic 5-HT_2B_ receptor.^147^ Furthermore, OS plays a crucial role in the emergence of depression, including by elevating the levels of ROS and NO in the mitochondrion of astrocytes.^253^ Proinflammatory signaling pathways are activated as a result of elevated OS, the mitochondrial dysfunction results in an increased generation of ROS and NO.^137^ As well as, the pathogenesis of MDD are associated with the inflammatory-immune response, as shown by elevated levels of proinflammatory cytokines, mainly IL-1β, TNF-α, and IL-6.^141^ The expression of neural cell marker proteins in neural cells, including Cx30/43,^162^ GFAP,^167^ AQP4,^172^ and S100B,^177^ are all decreased under MDD pathological conditions. In brain, KYN is metabolized by microglia to the neurotoxic metabolite QUIN and by astrocytes to the beneficial metabolite kynurenic acid (KynA), thus, QUIN is increased and KynA is decreased in MDD patients’ brain.^141,254,255^ Recently, growing evidence support that the occurrence of MDD are the results of the correlational disorders from multiple systems or organs, not only limiting in brain.^227,228^ The comorbidities of MDD have attracted widespread attention, the intestinal gut microbial dysbiosis, liver dysfunction, immune system disorders all play important roles in the pathogenesis of MDD. Stressful conditions can affect the gut microbiota, which in turn induces the production of inflammatory mediators (mainly IL-6 and IFN-γ).^256^ Proinflammatory cytokines or toxic QUIN resulting from alterations in the microbiota may pass through the BBB and activate NMDARs.^243^ Under the dysfunction of liver, the level of ammonia is increased in the brain.^257^ The pathogenic factors of various organs at the body level and the pathological changes of glial cells at the cellular level should attract more attention to explain the pathogenesis of MDD.

## New diagnostic approaches

MDD is a prevalent psychiatric disorder worldwide and is expected to become one of top disease in terms of burden by 2030.^258^ However, the current clinical diagnostic criteria for MDD are subjective, and diagnoses are mainly based on clinical symptoms, leading to high rates of missed and incorrect diagnoses. This section summarizes the newest research on diagnostic approaches for MDD, including serum indicators, neuroimaging indicators and multimodality scales. Research on new diagnostic approaches for MDD has the potential to improve our understanding of MDD pathogenesis and the accuracy of clinical diagnosis.

### Potential serum indicators

The pathological mechanism of MDD can be studied in two ways: by exploring the pathophysiology of the disease and by identifying MDD-related neurobiological indicators4. Hence, identifying potential biomarkers for MDD could allow accurate diagnosis, faster treatment and effective monitoring of the disease. Recently, an increasing number of studies have confirmed the involvement of OS and neuroinflammation in MDD pathology.^259,260^ Two novel biomarkers, serum nicotinamide adenine dinucleotide phosphate oxidase 1 (NOX1) and Raftlin, are reported to have good diagnostic value in MDD patients. The effectiveness of elevated NOX1 and Raftlin levels in diagnosing MDD has been evaluated in clinical trials; the related mechanism is that NOX1 can regulate the ROS-antioxidant balance in patients with MDD through OS and the inflammatory repsonse.^261^ The serum level of the chemokine-like protein TAFA-5 (FAM19A5) has also been reported to be increased in patients with MDD, and increased serum FAM19A5 levels are associated with reactive astrogliosis, neuroinflammation, and neurodegeneration.^262^ In addition, the level of serum FAM19A5 was shown to have a negative correlation with cortical thickness in specific brain regions. These findings suggest that serum FAM19A5 could be a potential biomarker for neurodegenerative changes in MDD.

### Functional magnetic resonance imaging indicators

In addition to serum indicators, neuroimaging metrics are potential objective tools for improving the accuracy of MDD diagnosis and must be studied in death. In recent years, many researchers have tried to diagnose MDD using MRI by identifying disease-specific functional and/or structural abnormalities in patients with MDD compared with healthy subjects.^263^ Structural MRI techniques, such as voxel-based morphometry (VBM), can be used to detect volume changes in gray matter.^264^ It has been reported that abnormal gray matter volume (GMV) in several brain regions is positively correlated with MDD.^265,266^ Regarding functional MRI, recent studies have revealed that cerebral functional abnormalities are not limited to specific brain regions in patients with MDD. These differences are also associated with hypoconnectivity within the frontoparietal network (FN), the DMN, and midline cortical regions.^267,268^ Furthermore, resting-state functional magnetic resonance imaging (R-fMRI) is an emerging neuroimaging technique used to study functional connectivity in the brain and holds great potential in aiding clinical diagnosis.^269^ It has the benefits of being noninvasive and easy to perform and offering high temporal and spatial resolution.^270^ As a result, it has played a significant role in MDD research and is a superior technique for researching MDD pathogenesis and identifying neuroimaging markers for MDD.^271^ Thus, indicators such as amplitude of low-frequency fluctuation (ALFF), fractional amplitude of low-frequency fluctuation (fALFF), regional homogeneity and functional connectivity (FC) have shown promise as neuroimaging markers for MDD. Recently, a study reported that increased average values of ALFF and fALFF in the right caudate and corpus callosum may serve as potential markers for diagnosing MDD.^272^ Another study based on the largest R-fMRI database of MDD patients confirmed that the DMN plays a crucial role in MDD diagnosis, as DMN FC is reduced in patients with recurrent MDD.^273^ These findings also suggest that the DMN should continue to be a prominent focus of MDD research.

### New multi-modal evaluation scales

Given that structural and functional abnormalities are associated with MDD,^274^ using multimodal approaches is more appropriate than relying on a single feature for the diagnosis of MDD. However, research results related to the effectiveness of neuroimaging techniques in diagnosing MDD remain inconsistent.^275^ This may be attributed to variations in the types of structural and functional features examined; however, more importantly, very few studies have used multimodal approaches to diagnose MDD.^276^ Recently, in a study utilizing multimodal MRI data, patients with MDD were successfully distinguished from healthy controls by radiomics analysis.^276^ Radiomics is a rapidly developing field involving the extraction of quantitative information from diagnostic images, and it can be mainly divided into three steps: image acquisition, analysis and model building.^277^ Additionally, omics and neuroimaging techniques can be combined to construct models for diagnosing MDD; specifically, 5-hydroxytryptamine receptor 1 A/1B methylation data can be integrated with resting-state functional connectivity (rsFC) data. It was shown that this combination could be used to more accurately distinguish patients with MDD from healthy subjects than R-fMRI data or DNA methylation data alone.^278^

By now, the widely accepted objective diagnostic indicators or methods for MDD are still deficient. In addition to the unclear pathogenesis of MDD, insufficient sensitivity and accuracy of detection instruments are also the main reasons, especially the correlation between imaging characterization and disease-specific changes that need to be discussed.

## Preventing the occurrence and recurrence of MDD

MDD is a disease with a high prevalence worldwide,^279^ and preventing its occurrence and recurrence is crucial. Lifestyle medicine is an evolving medical specialty that aims to prevent chronic, noncommunicable diseases through lifestyle interventions. The goal of lifestyle medicine is to prevent the occurrence and recurrence of disease by improving sleep hygiene and diet, increasing physical exercise, avoiding sedentary behavior, increasing social support, and improving mood. In recent years, an increasing number of studies have demonstrated that the occurrence and recurrence of MDD can be prevented by means of lifestyle medicine;^280^ we summarize these reports in this section.

### Sleep improvements

Improving sleep is an important strategy to prevent the occurrence of depression. Insomnia is included in the diagnostic criteria for MDD.^281^ However, few studies have examined whether treating insomnia can prevent the exacerbation of depressive symptoms. Treating insomnia can prevent the worsening of depressive symptoms, and cognitive behavioral therapy for insomnia (CBT-I) is a recommended intervention for treating insomnia to improve sleep and mood.^282–284^ As a first-line treatment for insomnia, CBT-I includes cognitive therapy, stimulus management, sleep restriction, improved sleep hygiene, and relaxation.^282,285^ CBT-I can also lead to sustained remission of insomnia-related disorders, and continuous treatment of insomnia with CBT-I can also reduce the occurrence and recurrence of MDD.^286^ Circadian rhythm support (CRS) can strengthen the circadian rhythm by means of scheduled bright light exposure, physical activity, and body warming.^287^ Although CRS has been reported to have only an indirect effect in alleviating sleep disturbance and depressive symptoms,^288^ treatment with CRS may help maintain the beneficial effects of CBT-I.^288,289^ In one study, 44% of untreated patients but 38%, 28% and 9% of patients treated with CRS, CBT-I, and CBT-I + CRS, respectively, experienced clinically significant worsening of depressive symptoms during a 1-year follow-up period. Between-group comparisons showed that the percentage of patients who experienced worsening of depressive symptoms was significantly different between the CBT-I + CRS group and the nontreated and CRS groups.^289^ In a randomized controlled trial, exacerbation of depressive symptoms over one year was decreased in insomia patients with an increased risk of depression and insomnia patients treated by therapist-guided CBT-I combined with CRS; however, untreated insomnia patients with a high risk of depression experienced clinically significant worsening of depressive symptoms.^288,289^

Disrupted sleep is a common symptom of depressive episodes and increases the risk of MDD,^290^ but the correlation between the onset of sleep disturbance and MDD is still unclear. Additionally, patients with symptoms of sleep disturbance have a greater risk of MDD occurrence and recurrence.^290,291^ One study suggests that disrupted sleep may affect monoamine function and the HPA axis,^292^ even causing hyperarousal and inflammation.^293^ Additional studies on the pathological mechanism of depression have suggested that the HPA axis is hyperactive in MDD patients and that sensitivity to negative feedback is decreased.^15^ Additionally, one prospective cohort study reported that a history of sleep disorders can increase the risk of depression later in life and that subjective sleep problems are associated with clinically significant depressive symptoms.^294^

### Dietary adjustment

Dietary adjustment is an effective, safe, and widely applicable method for preventing MDD, especially by inhibiting MDD-related pathological inflammation.^295^ Various nutrients can possess different anti-inflammatory properties; in contrast, there are many proinflammatory foods, such as those high in refined starch, sugar, and saturated fat and low in fiber and omega-3 fatty acids,^296^ which can promote the occurrence of inflammation to increase the risk of MDD.^297^ One study reported that the chance of being diagnosed with depression is higher among individuals who consume a proinflammtory diet than among those who consume an anti-inflammatory diet.^295^ Stimulation of the innate immune system by proinflammatory foods can result in mild inflammation and chronic illness, which may contribute to an increased risk of MDD.^298^ Furthermore, an increasing number of studies suggest that at the molecular and cellular levels, dietary factors have effects on neuronal function and synaptic plasticity, which may be implicated in the etiology of MDD.^299,300^ Therefore, adherence to a healthier diet can reduce the incidence of MDD, which is of great significance for the clinical treatment and prevention of depression.^295^

In addition, an increasing number of studies have identified the importance of the interaction among the microbiota, gut permeability, and immune-inflammatory processes in the pathophysiology of MDD.^301^ Because the interaction of bacteria of some taxa in the gut with peripheral inflammation with the brain may be related to depression pathophysiology,^302,303^ regulating the gut-microbe-brain axis may be a therapeutic and preventive strategy for psychiatric disorders.^304^ Restoration of the gut eubiosis can prevent the occurrence of MDD, and probiotics can normalize the gut ecosystem. Additionally, by altering the microbiota and regulating gut permeability, a gluten-free diet can alter the activity of the gut-microbe-brain axis, which has been discovered to be related to the pathogenesis of MDD.^305–307^ Other studies report that consuming a gluten-free diet and probiotic supplements together may inhibit the immune-inflammatory cascade in MDD patients, and decreased inflammation can improve the integrity of the gut barrier and alleviate depressive symptoms.^307^ Similarly, dietary fiber can also improve immune function by regulating the gut microbiota to prevent the occurrence of MDD,^308^ which is attributed to the inhibition of OS and inflammation.

### Exercise

Increasing evidence suggests that physical exercise can prevent some mental disorders in addition to cardiovascular disease.^280,309^ This finding suggests that physical exercise may be able to prevent MDD. As reported in some studies, physical exercise can effectively prevent depression by affecting many molecular and cellular pathways; for instance, physical exercise can stimulate VEGF expression,^310,311^ leading to cellular level changes, such as stimulation of angiogenesis, increased delivery of neurotrophic factors and oxygen by the vascular system,^312^ an increase in the neurogenesis rate and induction of synaptogenesis.^312,313^ Ultimately, VEGF improves function in the hippocampus, which is one of the brain regions related to depression and stress regulation.^314–316^ Exercise also reduces the levels of proinflammatory factors (e.g., IL-6) and increases the levels of anti-inflammatory factors (e.g., IL-10), which is beneficial for preventing the occurrence of MDD.^317–319^ Furthermore, physical exercise for approximately 45 minutes per day can significantly reduce the risk of MDD.^320,321^ High-intensity activity, such as aerobic exercise, dancing, and the usage of exercise machines, and low-intensity exercises, including yoga and stretching, can all reduce the occurrence of MDD.^322^ Specifically, the combination of aerobic exercise and stretching as a multimodal therapeutic strategies has a significant antidepressant effect in depressed inpatients.^323^

Patients with MDD have significantly more sedentary than ordinary people, and they engage in less physical activity than what is recommended, i.e., an average of 150 min of moderate- to high-intensity physical activity weekly.^324^ This finding suggests that decreasing sedentary behavior or increasing physical activity levels should be a priority to prevent the occurrence of disease. In psychiatric centers, aerobic exercise has received increasing attention as a valuable method of prevention.^324^ Studies report that reduced depressive symptoms in MDD patients can be observed after increasing aerobic exercise and stretching exercise, with more significant alleviation of depressive symptoms after 8 weeks of aerobic exercise.^325^ Reward positivity (RewP) and error-related negativity (ERN) were identified as potential biomarkers of the exercise treatment response in depression.^325^ In individuals with MDD, aerobic exercise was found to be beneficial in ameliorating depressive symptoms, particularly in those with more severe depressive symptoms and a higher baseline RewP.^325,326^ RewP may be useful for identifying those who will benefit from exercise as a treatment for depression.^325^

### Social intervention

Social support refers to the help provided by social relations and transactions.^327^ Social support may be obtained from a variety of individuals, including family members, friends, coworkers, and community members.^328^ Furthermore, a variety of factors, including the quantity and quality of support as well as subjectively perceived social support by individuals, impact the level of social support.^329^ It has been reported that MDD patients often lack social support, and receiving adequate social support can confer greater resistance to stress and prevent the occurrence and recurrence of MDD.^330,331^ Low-functioning social support or self-perceived poor social support causes worse symptoms and treatment outcomes in depressed patients.^332–334^ A previous study also reported that patients who lack adequate social support are more likely to experience MDD.^335^ Social support may have an influence on depression through neuroendocrine pathways,^336,337^ and social support can improve a person’s psychological wellbeing and make the individual more resistant to stress.^337^

Studies on structural social support, social network size, and mental health disorders have shown that less social contact and loneliness can cause more severe depressive symptoms.^338^ For individuals with MDD, it is necessary not only to increase the frequency of social contact but also to improve self-awareness and foster close functional supporttive relationships.^335,339^ Studies have reported that when controlling for all other variables, each aspect of social support is clearly associated with MDD, and to some extent, the occurrence of panic disorder in patients with MDD is more strongly associated with poor functional support. This finding suggests that functional support may be an important protective factor against MDD.^331,335^ Social support itself, especially emotional support,^340^ may alleviate and prevent depressive symptoms, and support from family members or friends can replace formal health care.^341^

**Fig. 5:**
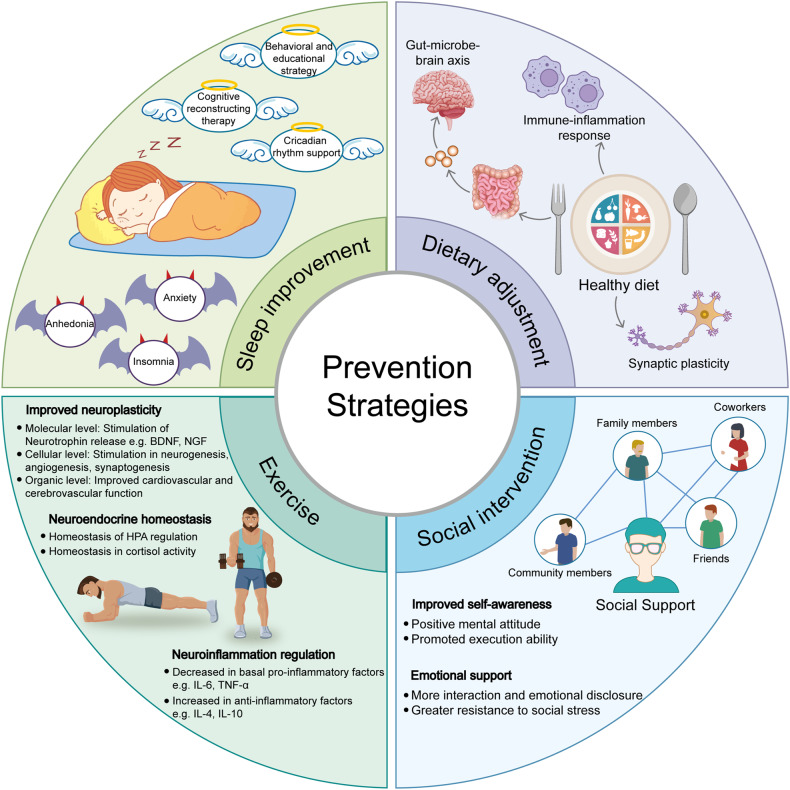
Schematic of prevention strategies for the occurrence and reoccurrence of MDD. An outline of various prevention strategies for MDD includes sleep improvement, dietary adjustment, exercise, and social intervention. Sleep disturbances have the high relationship with the occurrence of MDD, the anhedonia, anxiety and insomnia are the main symptoms of patients with MDD. The behavioral and educational strategies, cognitive reconstructing therapy and circadian rhythm support can be applied to improve sleep quality.^281,289^ Dietary adjustments are also suggested to have the potential effects to prevent the occurrence or re-occurrence of MDD, the improvement mechanism of diet may involve in the regulated immune-inflammatory responses, the improved gut-microbe-brain axis and synaptic plasticity.^295,299,304^ In addition, xxercise is an effective way to improve neuroplasticity, to maintain neuroendocrine homeostasis, and to regulate neuroinflammation, in order to effectively prevent the occurrence or re-occurrence of MDD.^280,309^ Importantly, getting social support from family members, friends, coworkers and community members can be helpful for the MDD patients’ recovery, these social interventions can let patients get emotional support and improve their self-awareness.^328,340^ Adobe Illustrator was used to generate this figure

In general, the pathological development of MDD is a gradual transition from subclinical state to clinical pathological changes. It is crucial to identify the core targets that lead to pathological changes from quantitative to qualitative changes during this process, and the above preventive interventions, sleep improvement, physical exercise, dietary regulation, and social intervention, may prolong or reverse the subclinical pathological stage (Fig. 5).

## Therapeutic drugs and strategies

This section summarizes new advances in research on the pharmacological mechanisms of common antidepressants and novel therapeutic strategies. Moreover, as laboratory animal models of MDD and other mental diseases are lacking, hindering the development of strategies for evaluating pharmacological effects and studying pathological mechanisms, we also discuss recent research on animal models.

### The molecular mechanism of antidepressants

#### Tricyclic antidepressants

In the late 1950s, the first TCAs were approved and used for the treatment of depression.^342^ TCAs have a common three-ring chemical structure, and the main TCAs are imipramine, amitriptyline, clomipramine, desipramine and doxepin. The pharmacological mechanism of TCAs mainly involves its interaction with neurotransmitters in the brain, resulting in changes in neurotransmitter levels and an antidepressant effect. First, TCAs can inhibit the reuptake of neurotransmitters, leading to antidepressant effects. For example, they can influence the levels of 5-HT, NE, and to a lesser degree, DA, causing an increase in neurotransmitter concentrations in the synaptic gap and increasing neurotransmitter signaling to exert pharmacological effects.^343^ However, different TCAs inhibit 5-HT and NE reuptake to varying degrees. For instance, amitriptyline, imipramine, and desipramine strongly inhibit 5-HT reuptake,^344^ clomipramine specifically inhibits NE reuptake, and nortriptyline can inhibit both NE and 5-HT reuptake while also exerting central anticholinergic effects.^345–347^ Additionally, TCAs can antagonize 5-HT_2A_ and 5-HT_2C_, thereby increasing the release of NE and DA in cortical areas.^348–350^ TCAs can bind to histamine receptors, especially H1 receptors, as well.^351^ By blocking H1 receptors, they can induce sedation and drowsiness, which may benefit depressed patients with sleep disorders.^352^ Furthermore, TCAs can also block muscarinic acetylcholine receptors, exerting anticholinergic effects and resulting in side effects such as dry mouth and constipation.^353^

In addition to the above-known pharmacological mechanisms, some recent studies have reported that amitriptyline can induce the activation of fibroblast growth factor receptor (FGFR), leading to the production of GDNF.^354^ In addition, amitriptyline can increase the expression of Cx43 to promote gap junction intercellular communication (GJIC) between astrocytes, thereby relieving depressive symptoms.^355^ This suggests that TCAs may also ameliorate severe depression through additional mechanisms involving astrocytes that are independent of the monoamine system to some extent. Further exploration is needed to fully understand the specific mechanism. Another study demonstrated that FKBP51, a crucial modulator of the glucocorticoid receptor (GR) pathway, can bind to clomipramine and impede its interaction with PIAS4. Inhibition of this interaction subsequently hinders sumoylation; this alteration represents a newly discovered mechanism by which the antidepressant drug exerts its effect.^356^

#### Selective serotonin reuptake inhibitor

According to a study, most severe depression patients are still advised to consider SSRIs as the initial choice for treatment.^350^ The main representative SSRIs drugs include fluoxetine, sertraline, paroxetine, and escitalopram. The mechanisms of action of SSRIs are commonly known as follows: first, SSRIs can selectively inhibit SERT, inhibiting the reuptake of 5-HT in the synaptic cleft and thereby exerting pharmacological effects.^357^ Second, SSRIs can impact the 5-HT signaling pathway, activating 5-HT_1A._^358,359^ In addition, studies have shown that antagonism of 5-HT_2A/2C_ receptors can enhance the effects of SSRIs such as fluoxetine.^360,361^ Third, long-term use of SSRIs can increase 5-HT transmission in the LC,^362^ thereby increasing the release of GABA to exert inhibitory effects on NA neurons.^363^ Fourth, long-term use of SSRIs is associated with neuroplasticity and neurogenesis in certain brain regions.^364^ SSRIs have been found to increase the expression of BDNF, a protein crucial for neuronal growth and survival, by acting on TrkB,^365^ which may contribute to the long-term therapeutic effects of SSRIs. Thus, our previous reports and others researches all suggested that astrocytic 5-HT_2B_ receptors may be the potential pharmacological target of SSIRs.^59,60,366–368^

According to previous studies by our group, in the absence of SERT, SSRIs such as fluoxetine can act as direct agonists of astrocytic 5-HT_2B_ receptors to exert antidepressant-like effects.^60,64,179,366,369^ In astroglia isolated from mice exposed to CUMS, fluoxetine activates the 5-HT_2B_ receptor, promoting ERK_1/2_ phosphorylation. This increases downstream c-Fos expression, which in turn boosts BDNF synthesis.^147^ Furthermore, administration of fluoxetine effectively inhibits SD-induced stimulation of the NLRP3 inflammasome by the AKT/STAT3 and ERK/STAT3 pathways in vivo, and SD dramatically triggers depressive-like behaviors by stimulating astrocytic P2X_7_Rs.^41,155^ As previously mentioned, leptin may increase the expression of the 5-HT_2B_ receptor in astrocytes via the LepR/JAK2/STAT3 pathway, and fluoxetine may be more effective in increasing BDNF levels and alleviating depressive-like behaviors due to the leptin-mediated increase in 5-HT_2B_ receptor expression.^130^ Both in vivo and in vitro, fluoxetine’s inhibitory actions on A1 reactive astrocytes depend on astrocytic 5-HT_2B_R.^55^ Recently, fluoxetine was shown to act as a 5-HT_2B_ agonist, and this finding is also supported by research by other groups. Fluoxetine has been reported to suppress the activation of A1 reactive astrocytes and decrease unusual behaviors in CMS-exposed mice. In vitro, Gq protein and b-arrestin1 are not necessary for fluoxetine’s effects on A1 astrocyte activation, and downstream signaling through astrocytic 5-HT_2B_R is responsible for fluoxetine’s inhibitory effects on A1 astrocyte activation in primary culture.^55^

#### Serotonin/norepinephrine reuptake inhibitors

SNRIs are often recommended as the initial choice for the treatment of MDD. Representative SNRIs include milnacipran, DXT, DVS, and venlafaxine. The molecular mechanisms of SNRIs can be summarized as follows: First, SNRIs inhibit the norepinephrine transporter (NET), which prevents the reuptake of NE into presynaptic neurons, leading to an increased concentration of NE in the synaptic cleft.^370^ Second, similar to SSRIs, SNRIs also inhibit SERT, resulting in an increased concentration of 5-HT in the synaptic cleft.^371^ For example, paroxetine and venlafaxine can inhibit SERT and, to a lesser extent, NET.^372^ Third, SNRIs inhibit the reuptake of both NE and 5-HT; thus, they have a dual mechanism of action. This dual inhibitory effect is believed to contribute to the broader therapeutic effects of SNRIs compared to SSRIs.^373^ Chronic treatment with fluoxetine has been shown to increase the expression of Cx43 in the rat PFC, which further prevents the dysfunction of astrocytic gap junctions induced by CUS and reverses the depressive-like behaviors caused by gap junction blockade.^71^

In a randomized controlled trial, MRI scan were taken after treatment with duloxetine and desvenlafaxine, and the results showed that the thalamo-cortico-periaqueductal network, which is associated with the experience of pain, may be an important target of action of antidepressant drugs.^374^

#### New potential pharmacological targets

The abovementioned antidepressants have been utilized as clinical therapies for MDD, but it is difficult to elucidate the exact pharmacological mechanisms of every medicine due to delayed clinical efficacy, poor treatment response to some patients, and difficulty in effectively controlling the incidence of suicide. Recently, several pharmacological agents have been discovered as potential antidepressants.

Ketamine, a noncompetitive antagonist of the NMDAR, has been shown to induce rapid and significant antidepressant effects within a few hours.^375^ Due to the rapid antidepressant effects of ketamine, unlike the delayed effects of traditional antidepressant drugs,^376^ research on this drug has continued and has revealed its mechanisms of action and potential drug targets. Ketamine can increase the level of BDNF in the prefrontal cortex, especially in the hippocampus, to exert antidepressant-like effects.^377^ Studies have suggested that ketamine can increase the synthesis of synaptic proteins through BDNF signaling dependent on the activate protein kinase B (Akt) and mammalian target of rapamycin complex 1 (mTORC1) signaling cascades.^378,379^ Ketamine may induce the activation of mTOR by the upstream kinase Akt, regulate the phosphorylation of GSK-3β, and exert antidepressant effects.^380^ Ketamine can block NMDARs in postsynaptic principal neurons in the PFC and hippocampus, increase synaptic function through homeostatic mechanisms, and reverse synaptic defects caused by chronic stress.^381,382^ Furthermore, by inhibiting NMDARs, ketamine can reduce the excitation of specific cortical GABAergic interneurons, resulting in a temporary increase in glutamate release that stimulates postsynaptic AMPA glutamate receptors. This, in turn, leads to the release of BDNF, activation of the TrkB receptor, and subsequent activation of the Akt/mTORC1 signaling pathway. These molecular events ultimately contribute to an increase in the number and functionality of synapses, leading to amelioration of depressive symptoms.^383^

Similar as ketamine, some other psychedelics can also produce fast and persistent antidepressant effects.^384^ Psilocybin, a classical psychedelic, can play its antidepressant roles by activating 5-HT_2A_ receptors (5-HT_2A_R).^385^ Thus, to block the 5-HT_2A_R can not produce the antidepressant effects of psilocybin, only induce the hallucinogenic-like behaviors in mice.^386^ This proposes 5-HT_2A_R may not be the real pharmacological target for its antidepressant effects. Another study reports that the combination of lysergic acid diethylamide (LSD) and psilocybin may exert long-term antidepressant effects by promoting neural plasticity, which dose not involve in the hallucinogenic effects.^384^ Additionally, to target 5-HT_2A_R, the combination of LSD and psilocybin can lead to biased activation of the mediated signaling pathway and produce antidepressant effects without the side effects of hallucinations.^387^ Thus, the administration of psilocybin can rapidly and persistently induce neuronal dendritic remodeling in the medial frontal cortex of mice, and the psilocybin-induced newly formed dendritic spines can successfully transform functional synapses, suggesting that synaptic rewiring may also be one pharmacological mechanism of the rapid antidepressant effects of psilocybin.^388^ To further dissociate the hallucinogens effects from the psychedelics can be beneficial to develop more specific antidepressants with better therapeutic capacities.

Additionally, some novel potential therapeutic targets for MDD have also been reported, such as TGF-β1^389^ and growth-associated protein 43 (GAP-43).^390^ Multiple studies have shown that antidepressants may cause changes in TGF-β1 expression. Fluoxetine, paroxetine, venlafaxine, and sertraline have been shown to have the potential to increase the levels of TGF-β1, which may contribute to their antidepressant effects.^391,392^ Venlafaxine has also been reported to exert neuroprotection by increasing the production of FGF-2 and TGF-β1 in astrocytes following stroke.^72^ Then, chronic administration of desipramine has been shown to upregulate the expression of GAP-43 in the hippocampus of rats, potentially influencing neuronal plasticity in the CNS.^390^ GAP-43 has been suggested as a relevant target for the pharmacological effects of antidepressants.^393,394^

**Fig. 6:**
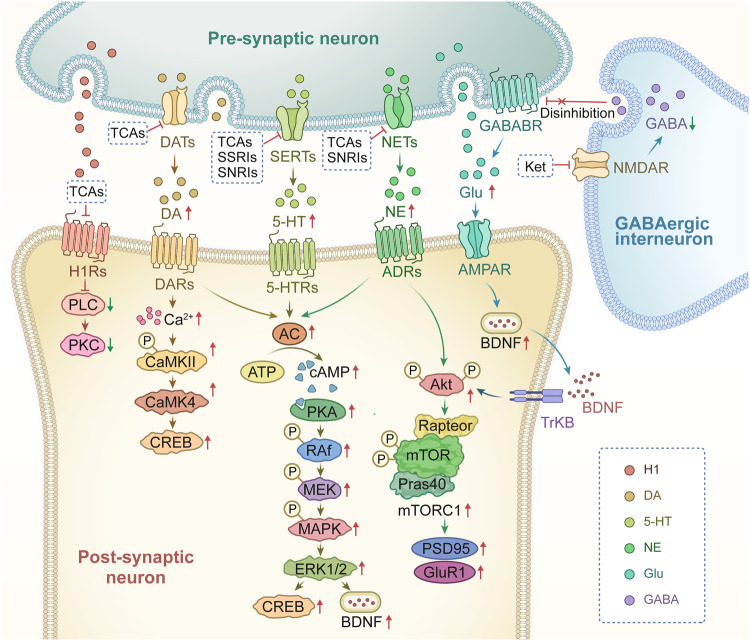
The molecular mechanisms of tricyclic antidepressants (TCAs), selective serotonin reuptake inhibitors (SSRIs), serotonin/norepinephrine reuptake inhibitors (SNRIs) and ketamine. TCAs can inhibit the protein kinase C (PKC) pathway by blocking the H1 receptors (H1Rs),^351,352^ TCAs decreases the reuptake of dopamine (DA) by inhibiting dopamine transporters (DATs) in the presynaptic membrane, and increases the DA concentration in the synaptic gap, increase the effect of DA on dopamine receptors (DARs) of postsynaptic membrane.^343^ The activated DARs increase Ca^2+^ dependent CaMKII and CaMK4, as well as, the secretion of CREB.^498,499^ In another way, the stimulated DARs by DA can also activate the cAMP-PKA pathway, which in turn activates the levels of CREB and BDNF by stimulating MAPK/ERK_1/2_ pathway.^500^ TCAs, SSRIs, and SNRIs can all inhibit the reuptake of 5-HT by SERTs, specially SSRIs have the selective inhibition on SERTs, which increase the concentration of 5-HT in the synaptic gap and play antidepressive roles by effecting on 5-HTRs in postsynaptic membrane,^343,344^ which also activate the cAMP-PKA pathway.^49,501^ Moreover, TCAs and SNRIs can also inhibit the reuptake of NE by NETs, which also increase the concentration of NE in the synaptic gap, and in turn activate the effect of NE on adrenoceptors (ADRs) and activate the cAMP-PKA pathway in postsynaptic membrane.^502^ Besides of the AC/cAMP/PKA pathway, the effect of NE on ADRs can also activate protein kinase B (Akt) phosphorylation and mammalian target of rapamycin complex 1 (mTORC1) by stimulating TrkB, in order to promote the secretion of postsynaptic density 95 (PSD95) and glutamate receptor 1 (GluR1).^502^ Ketamine works as the antagonist of NMDAR on GABAergic interneurons, it suppresses the excitation of subsets of GABAergic interneurons, which reduces the gamma aminobutyric acid (GABA) effects on gamma aminobutyric acid type B receptor (GABABR), and relieves the inhibition of GABAergic interneurons on the release of glutamate, the latter further stimulates AMPAR on postsynaptic membrane and increases the level of BDNF, even the release of BDNF stimulates the above TrkB/AKT/mTORC1 pathway.^503,504^ Adobe Illustrator was used to generate this figure

The most of above antidepressants have been widely used for the MDD patients according to the respective potential pharmacological actions (Fig. 6). Thus, the exactly neuromolecular mechanisms require deep studied and the new potential therapeutic targets and strategies still need further exploration.

### Novel therapeutic strategies

#### New animal models

**Fig. 7:**
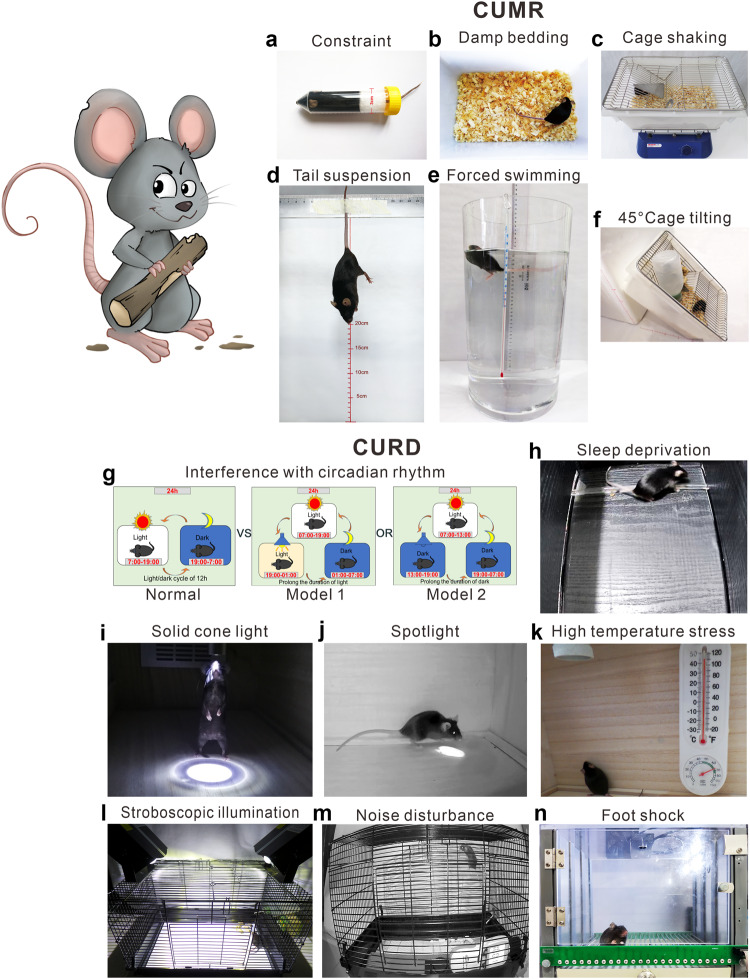
The protocol and stressors used for CURD and CUMR. In order to establish the CUMR model, a combination of various stressors includes interference of constraint (**a**), damp bedding (**b**), cage shaking (**c**), tail suspension (**d**), forced swimming (**e**), and cage tilting (**f**). Among these six stressors, two were randomly selected and administered daily for a duration of 3 weeks. On the other hand, to establish the CUMR model, a set of behavioral constraints includes circadian rhythm (**g**), sleep deprivation (**h**), interference of cone light (**i**), interference of followed spotlight (**j**), high temperature stress (**k**), stroboscopic illumination (**l**), noise disturbance (**m**), and foot shock (**n**). Similarly, two out of these eight constraints were randomly chosen and applied daily for a period of 3 weeks^62^

Establishing animal models with pathological features representative of those seen in humans is key for advancing MDD research. Currently, the widely utilized animal models of MDD include CUMS, behavioral despair (BD), learned helplessness (LH), and CSDS, drug withdrawal, and transgenic animal models.^395^ The CUMS model, one of the most commonly used animal models for MDD,^64,172^ exhibits depressive-like behaviors.^396,397^ According to a meta-analysis of 408 papers involving stress protocols, the most commonly used stressors for CUMS models are food and water deprivation, light cycle modification, wet bedding, cage tilting, social stress, and forced swimming.^398^ Recently, we constructed an improved depression model named the chronic unpredictable mild restraint (CUMR) model by using environmental interference.^62^ The stressors used to construct this CUMR mouse model included activity restriction, damp bedding, cage shaking, tail suspension, forced swimming, and 45° cage tilting. These stressors all restrict the activity of the mice; moreover, stressors that disturb physiological rhythms, chronic unpredictable rhythm disturbance (CURD), can cause manic-like behaviors in mice (Fig. 7). The disease-related pathological changes and serum indicators in the CUMR and CURD models are highly similar to those in patients in the clinic, and therapeutic medicines can effectively improve brain function and behavior in these models.^62^

#### Phototherapy

Phototherapy plays a significant role in regulating emotional behavior^399^ and can have strong and rapid effects on mood and alertness.^400–402^ There is increasing evidence for the therapeutic efficacy of phototherapy for MDD.^403,404^ The combination of phototherapy and antidepressants has better effects than antidepressants alone.^402,405^ Phototherapy utilizes bright light with a specific wavelength to stimulate the retina and affect the production of 5-HT and hormones in the brain.^406^ Furthermore, phototherapy can alleviate depressive-like behavior by targeting the retinal-thalamic ventral lateral geniculate nucleus/intergeniculate leaflet-lateral habenula (retinal-vlGN/IGL-LHb) circuit; this mechanism may explain how phototherapy alleviates MDD.^407^

#### Repetitive transcranial magnetic stimulation

Repetitive transcranial magnetic stimulation (rTMS) is an effective method used in clinical practice for treating patients with MDD.^408^ Multiple evaluations and analyses have shown that rTMS can effectively treat MDD in patients from different age groups, including children and adolescents,^409,410^ adults,^411,412^ and elderly patients.^413,414^ It is suggested that early use of rTMS in the treatment of depression in elderly patients may yield better results.^415^ Furthermore, research has indicated that rTMS can effectively treat perinatal depression.^416^ Increasing evidence suggests that rTMS of the anterior stimulation site of the left dlPFC can yield optimal treatment outcomes.^417–419^ A randomized controlled trial demonstrated that the efficacy of rTMS in treating depression is linked to precise targeting of the dlPFC, the activity of which exhibits a negative correlation with subgenual cingulate cortex activity.^420^ Identifying the optimal site for stimulation may further enhance the ability of rTMS to treat depression.^421^ Recently, a retrospective study was conducted, which included 29 systematic evaluations and reanalyzed 15 meta-analyses to assess the effectiveness and safety of transcranial magnetic stimulation (TMS) for treating MDD in adults.^422^ The results of the study indicated significant variations in the efficacy of TMS for MDD across different settings and revealed poor tolerability in certain populations, the further research is necessary to identify specific beneficiary populations for TMS in treating MDD and to personalize treatment based on comprehensive and detailed information.^422^

#### Psychological intervention

MDD is characterized by a gradual onset and a high risk of relapse.^421^ The American Medical Association recommends psychological interventions for individuals who are at a high risk of MDD. Some of the interventions commonly used for depression treatment include acceptance and commitment therapy, cognitive therapy, cognitive behavioral therapy (CBT), interpersonal therapy, and psychodynamic therapies.^423^ Specifically, the combination of psychological interventions and antidepressants effectively decreases the risk of relapse in cases of MDD.^424–426^

#### Acupuncture

Acupuncture, which mainly includes traditional body needling, moxibustion, EA, and laser acupuncture, is a traditional Chinese treatment modality used to treat various diseases.^427^ Compared with pharmacological therapies, acupuncture is more cost-effective and has fewer side effects.^428^ EA stimulation can effectively treat MDD;^429–431^ however, the specific mechanism by which acupuncture treats depression remains unclear. In previous research, EA at the ST36 acupoint was shown to prevent shrinkage of the prefrontal cortical astrocytes and alleviate depressive-like behavior in mice exposed to CUMS.^432^ The results of an 8-week clinical study involving 46 female patients with severe depression suggested that acupuncture may achieve therapeutic effects by modulating the corticostriatal reward/motivation circuit in patients with severe depression.^433^ Additionally, studies indicate that EA may have the potential to promote neuronal regeneration and exert antidepressant effects by elevating the phosphorylation of cyclic adenosine monophosphate response element binding protein and the levels of BDNF.^434^ Acupuncture at the GV20 and GV24 acupoints may alleviate depression symptoms by regulating the calmodulin-dependent protein kinase (CaMK) signaling pathway.^435^ The antidepressant effect of EA may also be associated with increased synaptic transmission in the ventromedial prefrontal cortex (vmPFC).^436^ A recent meta-analysis of 43 randomized controlled trials involving adult subjects with acupuncture for MDD demonstrated that acupuncture, either alone or in combination with antidepressants, significantly reduced the hamilton depression scal scores and had fewer adverse effects compared to antidepressants, however, further rigorous experiments are still required to determine the optimal frequency of acupuncture for MDD in order to achieve better efficacy.^437^

In conclusion, the common antidepressants can improve some depressive symptoms in some patients with depression, but are always associated with the risk of adverse effects or recurrence. Although some new developed treatment methods can improve depression symptoms in a certain program, the compatibility between potential treatment mechanisms and pathological mechanisms still needs further research. In particular, the therapeutic principle of acupuncture still needs to be explored in depth, and the accompanied therapeutic mechanism and application potential of traditional Chinese medicine in depression deserve to be explored in depth.

## Clinical research progress

In summary, the pathological features of MDD and pharmacological mechanism of antidepressants have been widely studied. Furthermore, there have been many clinical studies on MDD, and studies of human postmortem tissues and clinical medical images, multomics studies, and preclinical/clinical trials of new therapeutic drugs have improved our understanding of the disease mechanism.

### Transcriptional studies of human postmortem tissue

A recent meta-analysis of eight transcriptome datasets identified 566 disease-related genes that are consistently up- or downregulated in patients with MDD. The brain regions in which these genes are expressed include the amygdala, subgenual anterior cingulate, and dorsolateral prefrontal cortex, and the associated molecular pathways include reduced neurotrophic support, neural signaling, and GABA function.^438^ Through the discovery of nonoverlapping proteins that bind to calcium parvalbumin, calretinin, and the neural peptide somatostatin, subgroups of GABA interneurons that govern main pyramidal neurons differently were identified.^439^ Decreased cortical levels of GABA and specific populations of GABA neurons have been reported in investigations of postmortem MDD patient tissues,^440^ and the SST mRNA level is specifically decreased in patients with MDD.^213^

The DR nucleus is the largest and most significant conduit of forebrain serotonergic input.^441^ In postmortem samples of the human brain, several transcriptional regulators are dysregulated within the DR, including transcription-related elements (such as EGR1, TOB1, and CSDA), which bind to genes to stimulate their expression directly or in response to environmental cues, and NRs (NR4A2, NR4A3, THRA, and THRB), which are activated by ligands and regulate translation by targeting genes.^442^ In addition, transporters for GRs generally regulate the activity of the HPA axis by negative feedback.^443^ According to studies of postmortem brain tissues, hyperactivity of the HPA axis in MDD patients could be caused by methylation-mediated changes in GR transcription.^444^ The expression of nerve growth factor-inducible protein A (NGFI-A), an enzyme that bindss exon 1 F of GR, is reduced in the hippocampus of patients with MDD, which may contribute to low methylation levels in the brain.^444^ Moreover, in postmortem MDD patients, total GR levels are unchanged, while level of GRα in the amygdala and cingulate gyrus is decreased.

### Sex-related molecular markers of MDD

Women are more likely than males to experience recurring MDD^445^ and are twice as likely to experience MDD throughout their lifetimes.^446^ Compared with male patients, female patients with MDD have symptoms that manifest sooner in the disease course, last longer, and are more severe; in addition, they experience hunger changes, weight fluctuations, and sleep difficulties more frequently.^447,448^

In postmortem samples of patients who committed suicide due to MDD, the expression of DNA methyltransferases (DNMTs) in the frontopolar cortex was found to be more significantly increased in women than in men; elevated methylation is associated with decreased levels of the GABA_A_ receptor alpha-1 subunit in men, which supports sex-related epigenetic alterations in transcription.^449^ A gene array meta-analysis also revealed sex differences in MDD, with depressed females being more likely than depressed men to have lower production of somatostatin, a GABA neuron biomarker in corticolimbic brain regions according to postmortem analysis.^450^ X-linked chromosomal polymorphisms affect the expression of the GABA-synthesizing enzyme and somatostatin.^450^ Analyses of postmortem brain tissues showed an increase in the transcription of numerous glutamate-related genes in the prefrontal cortex in depressed women but not in depressed men; depressed women exhibited more alterations in glutamate receptor expression, while depressed men showed only GRM5 downregulation.^451^

In postmortem brain specimens, there were no transcription differences between MDD men and controls, and the levels of 5-HT_1D_ receptors and the transcription factors NUDR and REST, which regulate 5-HT activity, in 5-HT-containing neurons in the ventral raphe nuclei were found to be higher in MDD females.^452^ 5-HT receptors and regulators were shown to exhibit sex-specific alterations in expression at the protein level, and postmortem investigations have largely focused on female subjects. The protein levels of 5-HT_1A_R and NUDR, which regulate 5-HT signaling, in the prefrontal cortex were found to be lower in MDD women than in control subjects; however, this difference was not observed in MDD males compared with controls.^453^ The NA/NE system, especially in the LC, is another monoaminergic system that exhibits sex-related variations and influences MDD risk. In fact, some researchers have found that the levels of microRNAs (miRNAs), short RNA molecules that control the expression of genes and play roles in psychological disorders,^454^ are higher in the LC of suicidal female subjects than in the LC of suicidal male subjects. MiR-1179 is associated with GRIA3 and MAOA, which are involved in neuropsychiatric diseases.^455^

OS is commonly linked to the onset of MDD. A study found that whereas cysteine and 1-methylinosine levels were much higher in males with MDD, they were significantly lower in females with MDD.^456^ These metabolites are related to OS. Furthermore, several studies found a significant link between MDD and lipid metabolism;^457^ for example, as 1-Oalkyl-2-acyl-PEs levels are decreased in MDD, showing a negative correlation with the extent of depression, lysophospholipid (LPC) and phospholipid (PC) levels are increased in MDD, exhibiting a substantial positive correlation with depression severity.^458^ Similarly, a study found that men and women had different lipid concentrations.^456^ These clinical data suggest that sex differences in MDD may result from differences in OS and lipid metabolism, but further research is required to make this connection.

### Multiomics studies

Transcriptome studies, which explore relationships among the expression of genes and diseases, are regarded as an essential for investigating disease-causing mutations in genes, the mechanisms of disease development and progression, and disease-related target genes.^459^ Dorsolateral prefrontal cortex tissues have been employed to identify genes and miRNAs that show changes in expression and biological processes that are altered in patients with MDD.^460^ Serpin Family H Member 1 (SERPINH1), IL-8, humanin like-8 (MTRNRL8), and chemokine ligand 4 (CCL4) are among the genes whose expression is altered in MDD.^460,461^ According to Gene Ontology (GO) enrichment analysis, MDD is related to decreased expression of genes related to oligodendrocyte development, glutamatergic neurotransmission modulation, and oxytocin receptor expression. These findings confirm that impairment of the blood-brain barrier and microglial, endothelial cell, ATPase, and astrocyte function exacerbate MDD; the involvement of these cells, molecules, and structures in MDD should be further investigated.^460^

The field of study known as genomics focuses on the transcription of genes, the precise interactions among genes, and the control of gene activity. MDD has been linked to numerous biological processes, including energy metabolism. When the transcription of genes involved in glycolysis and glycogen synthesis was examined in the hippocampus of depressed rats, it was found that the mRNA expression of Slc2a3, which codes for GLUT3, is considerably increased.^462^ Glyceraldehyde-3-phosphate dehydrogenase (GAPDH) and lactate dehydrogenase B (LDHB) mRNA levels were found to be substantially decreased in MDD.^462^ The transcription of genes in the brain tissues of IL18^-^/^-^ mice was examined with the use of genome-wide microarrays, and the results revealed that urocortin 3 (Ucn3) expression was increased.^463^ Ucn3 controls how the body processes glucose;^464^ therefore, a change in Ucn3 expression will result in energy imbalance. Gene comethylation analysis was performed in the brains of individuals with MDD. The findings revealed that the methylation of genes associated with mitochondria was dramatically decreased, indicating impaired mitochondrial function.^465^

Metabolomics has recently emerged as a useful technique for identifying markers and pathways associated with a wide range of diseases.^466^ It is often used to analyze the mechanisms underlying disease occurrence and progression and the effects of small-molecule compounds. In one study, targeted metabolomic analysis of the CSF of 14 MDD patients who were not taking medication, 14 MDD patients in remission, and 18 healthy controls was performed.^467^ An analysis of the tryptophan, tyrosine, purine pathways, and associated pathways revealed that in patients in remission, methionine levels were higher, while tryptophan and tyrosine levels were lower. The same group of patients also showed changes in the methionine-to-glutathione ratio, indicating alterations in OS and methylation. The levels of these same metabolites were altered in MDD patients who were not taking medication, although not to a significant degree.^467^

### Clinical medical imaging studies

MRI has been widely employed in research in recent years to pinpoint patterns of brain alterations linked to MDD. Many studies have demonstrated that structural and fMRI has outstanding potential as trustworthy imaging modalities for monitoring MDD treatment responses. A study indicated that MDD patients had large volume decreases in various frontal areas, such as the anterior cingulate cortex and OFC, which were linked to problems with stress management and emotional processing.^468^ People with MDD also exhibited structural changes in their parietal lobes.^469^ Alterations in the total gray matter volume and an increase in cortical thickness are the two findings that are most consistent.^470^

The functional changes in the frontal lobe in MDD are hotly contested. A study discovered lower precuneus, supragenual anterior cingulate cortex, dorsomedial PFC, and dorsomedial thalamus lower activity when processing pleasant stimuli in MDD patients.^471^ Another study found that during the processing of favorable self-indulgent information, individuals with MDD displayed higher activity in the medial PFC and anterior cingulate cortex.^472^ The right hippocampus, parahippocampal gyrus, left amygdala, and the whole caudate nucleus all had functional changes in activity in MDD patients compared to healthy controls, indicating that the temporal lobe might be involved in the pathogenesis of MDD.^473^

Although it is not feasible to evaluate synapse density directly in people in vivo, positron emission tomography (PET) can be utilized to gather useful information. It is thought that impairments of functional connections and synaptic atrophy are two factors that contribute to the symptoms of MDD. An indirect method of estimating synaptic density is to count the number of nerve terminals using synaptic vesicle glycoprotein 2 A (SV2A). The researchers examined synaptic density in MDD patients who were not taking any medication using positron emission PET with the SV2A radioligand [^11^C] UCB-J.^474^ The results revealed that reductions in the synapse density in areas connected with various processes, such as emotion control and thought (the dorsolateral prefrontal cortex, anterior cingulate cortex, and hippocampus), are related to to the severity of depressive disorders. Additionally, it was shown that compared with healthy subjects, subjects with MDD had reduced dlPFC resting-state connectivity throughout the brain. It was found that the dlPFC-posterior cingulate cortex connection was inversely negatively linked to the severity of depression symptoms and connected with synapse activity in the dlPFC, indicating that synaptic loss may impair antagonistism within the centers of both networks, which are typically at odds.^474^

### Preclinical and clinical trials of new therapeutic drugs

Esmethadone is a new, noncompetitive NMDAR antagonist^475^ that exhibits fast antidepressant-like action by improving performance of rats in the forced swim test.^476^ Esmethadone can also alleviate neural dysfunction linked to symptoms of depression by boosting the synapse and spine density and restoring spinogenesis, in addition to correcting depressive-like behaviors in animal models of depression.^378,477^ Esmethadone was found to reduce cognitive symptoms in individuals with MDD in a stage II clinical study^478^ and to increase the levels of circulating BDNF in normal individuals in a stage I clinical investigation.^479^ In a phase II study involving patients who had received insufficient benefit from conventional antidepressants, esmethadone demonstrated immediate, strong, and long-lasting antidepressant benefits.^478^

Ketamine is the most well-known rapid-acting antidepressant and an NMDAR antagonist.^383^ GluN1, GluN2, and GluN3 are NMDAR subunits.^480^ Ketamine exerts a quick and effective antidepressant effect by binding to the asparagine 616 residue of GluN1 and the leucine 642 residue of GluN2A.^192^ In a clinical experiment, the effect of supplementary injection of subanesthetic doses of ketamine on thoughts of suicide in MDD patients was evaluated, and the results showed that the reduction in thoughts of suicide among MDD patients receiving ketamine was mostly sustained.^481^ In several studies, a single dose of ketamine reduced immobility in the forced swim test immediately after injection and had effects similar to those of an antidepressant.^482,483^

The S-enantiomer of ketamine, esketamine, has been approved by the U.S. Food and Drug Administration (FDA) for depression treatment.^383^ Moreover, formulations of ketamine are also being developed, and intranasal esketamine spray has shown high efficacy in treating MDD.^484^ Additionally, hydroxynorketamine (HNK), a metabolite of ketamine, can exert its anti-depressive effects by an NMDAR-independent mechanism.^377^ One of these mechanisms involves increasing BDNF levels; an increasing number of studies have shown that BDNF signaling is an important target of antidepressants.^377^ Thus, ketamine can also exert anti-inflammatory effects, a large amount of evidence suggests a tight relationship between neuroinflammation and the pathogenesis of MDD.^485–487^ A summary of clinical trials related to new therapeutic drugs for MDD is shown in Table 1.

**Table 1:** Clinical trials of new therapeutic drugs for MDD

Study	Duration	Mean age (SD) in years	Mood disorder type	Diagnostic tool	Interventions	Control	Outcome indicators	Blinding of participants	Outcomes
Fava M et al.^478^	7 days	NR	MED, with an inadequate response to one to three courses of antidepressant treatment	DSM-5; HAMD	(1) REL-1017 75 mg (Day 1), 25 mg/day (Days 2-7) (2) REL-1017 100 mg (Day 1), 50 mg/day (Days 2-7)	Placebo	MADRS; SDQ; CGI-S; CGI-I	Double blind	REL-1017 may have rapid and sustained antidepressant effects in patients with inadequate response to antidepressant treatment.
De Martin S et al.^479^	10 days	39 (8)	Healthy	/	REL-1017 25 mg	Placebo	BDNF plasma levels; systolic BP; diastolic BP	Double blind	Administration of 25 mg of REL-1017 significantly increased BDNF plasma levels and significantly decreased diastolic blood pressure.
Hochschild A et al.^481^	2 days	38.4 (13.2)	MDE, unipolar depression	DSM-IV; HAMD-17; SSI	Ketamine 0.5 mg/kg	Midazolam 0.02 mg/kg	SSI; HAMD-24; POMS; BDI	Double blind	Ketamine resulted in greater improvements in HDRS, HDRS, BDI and POMS scores and reduced suicidal ideation in patients.
Daly EJ et al.^484^	10 weeks, with an additional 8 weeks of post-treatment follow-up	44.7 (10.0)	TRD	DSM-IV-TR	Esketamine 28 mg, 56 mg, or 84 mg twice weekly	Placebo, an inactive substance	MADRS	Double blind	Antidepressant effects of intranasal esketamine in the treatment of TRD are rapid and dose-related.
Abbasi SH et al.^488^	6 weeks	NR	MDD	DSM-IV-TR; HAMD-17	Celecoxib 200 mg twice daily plus sertraline 200 mg/day	Placebo plus sertraline 200 mg/day	HAMD; IL-6 concentrations in the sera	Double blind	The serum IL-6 concentration in the celecoxib group was significantly reduced, which may be related to its antidepressant activity and can be used as an auxiliary antidepressant drug.
Akhondzadeh S et al.^489^	6 weeks	34.6 (6.8)	MDD	DSM-IV-TR; HAMD	Celecoxib 400 mg/day plus fluoxetine 40 mg/day	Placebo plus fluoxetine 40 mg/day	HAMD	Double blind	Celecoxib combined with fluoxetine is more effective than fluoxetine alone in treating major depression. Celecoxib may be an effective adjunct to treatment of patients with major depressive disorder.
Nettis MA et al.^490^	4 weeks	47.0 (10.0)	MDD with peripheral inflammation (CRP ≥ 1 mg/L)	DSM-5; MINI; HAMD-17; levels of serum CRP	Minocycline 200 mg/day	Placebo	HAMD-17; BDI- II; CGI; PSS; SHAPS; STAI-S; STAI-T: levels of inflammatory biomarkers	Double blind	Add-on therapy with minocycline may be effective in patients with MDD in patients with low-grade inflammation and CRP ≥ 3 mg/L
Hasebe K et al.^491^	12 weeks	51.7 (14.4)	MDD	MINI-PLUS 5; MADRS	Minocycline 200 mg/day	Placebo	HAMA; Q-LES-Q-SF; LIFE-RIFT; PGI; CGI-I; levels of IL-6, LBP and BDNF in blood samples	Double blind	There were no overall changes in IL-6, LBP or BDNF following adjunctive minocycline treatment.
Su KP et al.^492^	2 weeks	53 (10)	Depression induced by IFN-α	DSM-IV	Omega-3 fatty acids: EPA 3.5 g/day or DHA 1.75 g/day	Placebo (high oleic oil)	HAMD-21; NTRS; percentage of participants with MDE induced by IFN-α	Double blind	EPA is effective in preventing depression in HCV patients receiving IFN-α.
Berk M et al.^493^	12 weeks	20.2 (2.6)	MDD	SCID-I/P; MADRS	Rosuvastatin 10 mg/day or aspirin 100 mg/day	Placebo	MADRS; QIDS-SR; GAD-7; CGI-I/S; PGI; Q-LES-Q-SF; SAS-SR; SOFAS	Triple blind	The addition of aspirin or rosuvastatin did not produce any beneficial effects in the treatment of depression in young adults, but rosuvastatin may have potential therapeutic role in adolescent depression.
Meltzer-Brody S et al.^494^	3 days, with an additional 4 weeks of post-treatment follow-up	NR	PPD	SCID-I; HAMD	Brexanolone 90 μg/kg/h or brexanolone 60 μg/kg/h	Placebo	HAMD-17; CGI-I; MADRS; EPDS; PHQ; GAD-7	Double blind	Compared with placebo, after 60 hours of intravenous infusion of brexanolone, the total HAMD score of patients with postpartum depression was significantly reduced, and the drug effect was rapid and long-lasting.
Leal GC et al.^495^	7 days	NR	TRD; and failure to respond to at least two adequate antidepressant trials in the current episode	MINI; DSM-5; MADRS	(*R*)-ketamine 0.5 mg/kg	Placebo (saline solution)	MADRS; CGI-S; CGI-I	Double blind	(*R*)-ketamine is capable of producing rapid and potent antidepressant effects in TRD subjects.

*SD* standard deviation, *NR* not reported, *MDE* major depressive episode, *DSM* Diagnostic and Statistical Manual of Mental Disorders, *HAMD* Hamilton Depression Scale, *MADRS* Montgomery-Asberg Depression Rating Scale, *SDQ* Symptoms of Depression Questionnaire, *CGI-S* Clinical Global Impressions Severity Scale, *CGI-I* Clinical Global Impressions Improvement Scale, *BDNF* brain-derived neurotrophic factor, *BP* blood pressure, *SSI* Beck Scale for Suicidal Ideation, *POMS* Profile of Mood States, *BDI* Beck Depression Inventory, *TRD* treatment resistant depression, *MDD* major depressive disorder, *IL-6* interleukin-6, *CRP* C-reactive protein, *MINI* Mini International Neuropsychiatric Interview, *PSS* Perceived Stress Scale, *SHAPS* Snaith–Hamilton Pleasure Scale, *STAI-S* Spielberger State-Trait Anxiety Rating Scale-State, *STAI-T* Spielberger State-Trait Anxiety Rating Scale-Trait, *HAMA* Hamilton Anxiety Scale, *Q-LES-Q-SF* Quality of Life Enjoyment and Satisfaction Questionnaire Short Form, *LIFE-RIFT* Range of Impaired Functioning Tool, *PGI* Patient Global Impression, *LBP* lipopolysaccharide binding protein, *IFN* interferon, *EPA* eicosapentaenoic acid, *DHA* docosahexaenoic acid, *NTRS* Neurotoxicity Rating Scale, *SCID* Structured Clinical Interview, *QIDS-SR* Quick Inventory of Depression Symptomatology–Self Report, *GAD-7* Generalised Anxiety Disorder seven-item scale, *SAS-SR* Social Adjustment Scale–Self Report, *SOFAS* Social and Occupational Functioning Scale, *PPD* postpartum depression, *EPDS* Edinburgh Postnatal Depression Scale, *PHQ* Patient Health Questionnaire

The development of the present therapeutic medicines in clinic mainly targets the discovered pharmacological targets, mainly focusing on the key receptors or enzymes. However, at the organelle level of neural cells, the disturbed energy metabolism of mitochondria and the related RNA drugs, as well as the dysfunctions of lipid and glucose metabolism in psychopathological condition, still need deep exploration. Totally, the research on the mechanism of therapeutic drugs always requires the development of pathological mechanisms as support.

## Conclusions and future perspectives

MDD is a heterogeneous disease, its pathological and pharmacological mechanisms are still unclear, and diagnostic and therapeutic methods for MDD are limited. SSRIs and SNRIs are the first-line treatments for MDD in the clinic; however, a sizable portion of MDD patients do not respond well to the currently available antidepressants. According to research on real-world sequential therapies, even after numerous treatment attempts, almost 30% of MDD patients do not experience remission. This suggests that the existing theories and hypotheses cannot completely explain the pathogenesis of MDD and that more research on the pharmacological mechanisms of currently available antidepressants is still needed. We mainly discussed the potential etiology and pathogenesis of MDD from the perspective of widely accepted theories, including the neurotransmitter and receptor hypothesis, HPA axis hypothesis, cytokine hypothesis, neuroplasticity hypothesis and systemic influence hypothesis. A more comprehensive understanding of the pathophysiological mechanisms of MDD might significantly improve our capacity to develop preventive and more effective therapeutic methods that can help reduce the burden of and pain caused by major depression. Knowledge of the cellular processes that drive these alterations and the symptoms they cause may offer crucial will provide insight for new treatments.

MDD is connected with several cellular and structural modifications in the nervous system. Nonetheless, in the majority of these alterations cannot be consistently observed in vivo. Therefore, several issues need to be considered in future research: (i) Studies of animal models have made important contributions to our understanding of the pathophysiology of major depression, and more representative animal models of MDD should be developed. (ii) Because of our incomplete understanding of the disease and the disease’s intrinsic intricacy, there is an urgent need to develop updated imaging technologies and imaging software to allow advances in our understanding of the disease. (iii) The therapeutic shortcomings of traditional antidepressants have prompted the need for further drug discovery and development. (iv) MDD is strongly associated with many systems, and it will be important to further elucidate the mechanisms associated with MDD and other pathological conditions.
